# Actomyosin-driven force patterning controls endocytosis at the immune synapse

**DOI:** 10.1038/s41467-019-10751-7

**Published:** 2019-06-28

**Authors:** Anita Kumari, Judith Pineau, Pablo J. Sáez, Mathieu Maurin, Danielle Lankar, Mabel San Roman, Katharina Hennig, Vanessa F. Boura, Raphael Voituriez, Mikael C. I. Karlsson, Martial Balland, Ana-Maria Lennon Dumenil, Paolo Pierobon

**Affiliations:** 1 Institut Curie, PSL Research University, INSERM U932, 26 rue d’Ulm, 75248 Paris, Cedex 05 France; 2 Université Paris Descartes, Paris, 75006 France; 3 Laboratoire Interdisciplinaire de Physique, Université Joseph Fourier (Grenoble 1), 38402 Saint, Martin d’Hères Cedex 9 France; 4 Department of Microbiology, Tumor and Cell Biology, Karolinska Institutet, Stockholm, 17177 Sweden; 5 Laboratoire de Physique Théorique de la Matière Condensée, UMR 7600 CNRS /UPMC and Laboratoire Jean Perrin, UMR 8237 CNRS /UPMC, 4 Place Jussieu, 75255 Paris, Cedex 05 France

**Keywords:** Endocytosis, Actin, Myosin, B-cell receptor

## Abstract

An important channel of cell-to-cell communication is direct contact. The immune synapse is a paradigmatic example of such type of interaction: it forms upon engagement of antigen receptors in lymphocytes by antigen-presenting cells and allows the local exchange of molecules and information. Although mechanics has been shown to play an important role in this process, how forces organize and impact on synapse function is unknown. We find that mechanical forces are spatio-temporally patterned at the immune synapse: global pulsatile myosin II-driven tangential forces are observed at the synapse periphery while localised forces generated by invadosome-like F-actin protrusions are detected at its centre. Noticeably, we observe that these force-producing actin protrusions constitute the main site of antigen extraction and endocytosis and require myosin II contractility to form. The interplay between global and local forces dictated by the organization of the actomyosin cytoskeleton therefore controls endocytosis at the immune synapse.

## Abstract (web summary)

The immune synapse promotes cellular information exchange but the role of biophysical forces in synapse function is unclear. Here, the authors show that B cells exert two types of forces, a centripetal myosin II-driven force and a central actin protrusive force at the site of antigen extraction.

## Introduction

Cells are endowed with the ability to internalize substrate-bound molecules, which they recognize through specific surface receptors. Although the role of substrate mechanics has been extensively investigated in the context of adhesion, its impact on receptor endocytosis remains unclear. A typical case of coupling between substrate mechanics and juxtacrine signaling (i.e., by direct contact) occurs at the immunological synapse, i.e., the tight contact zone that forms between a lymphocyte and an antigen-presenting cell^1,2^. In the case of B lymphocytes, formation of the immunological synapse results from the engagement of the B-cell-antigen receptor (BCR) by antigens exposed at the surface of neighboring cells in vivo. The immune synapse provides a platform that facilitates signaling and leads to antigen internalization^3–5^, which is needed for B cells to ultimately produce high-affinity antibodies and generate immune memory^6,7^. As endocytosis often involves surface-tethered rather than soluble molecules when occurring in tissues, antigen internalization at the B-cell synapse provides a valuable model to study the impact of mechanics in this process.

Different experimental systems have been developed as surrogate antigen-presenting cells to study antigen extraction at the B-cell synapse ex vivo: planar lipid bilayers^6^, plasma membrane sheets^8^, and polystyrene beads^9^. On lipid bilayers, the immune synapse consists of a set of concentric patterns in which molecules and cytoskeletal components are partitioned: a distal supramolecular antigen cluster (dSMAC) with an actin ring, a peripheral supramolecular antigen cluster (pSMAC) enriched for adhesion molecules and a central supramolecular antigen cluster (cSMAC) in which antigens concentrate^6^. The first antigen extraction model to be proposed was based on the observation that B cells spread over antigen-coated substrates and then contract, allowing the transport of BCR-bound antigens towards the cSMAC^10^. A second model arose from Atomic Force Microscopy experiments monitoring interactions between the BCR and plasma membrane sheet-bound antigens. These experiments showed that B cells internalize these antigens by actively pulling on BCR-antigen complexes^8^. Both these mechanical models rely on the actin-based molecular motor non-muscular myosin II. In the first model, myosin II generates a global actomyosin contraction that drives antigen transport towards the cSMAC, whereas in the second model, myosin II acts locally by pulling on individual BCR-antigen complexes. Intriguingly, punctuated actin structures have also been observed in mouse B cells, where they were found to colocalize with BCR microclusters, and in human B cells, where they were shown to be involved in BCR signaling and antigen extraction^11,12^. Whether and how these actin structures are related to myosin II activity is not understood.

Here we investigate the spatio-temporal organization of forces exerted by B lymphocytes during antigen extraction. We show that they display a stereotypical patterning profile that includes two components: (1) peripheral forces resulting from the centripetal flow of myosin II and (2) central forces exerted by local invadosome-like actin protrusions, which mediate antigen extraction. Noticeably, we find that these actin protrusions need myosin II-dependent peripheral forces to form, reconciling the models previously proposed for antigen extraction. We conclude that the interplay between global and local forces, governed by the dynamics of the actomyosin cytoskeleton, controls endocytosis at the immune synapse. Myosin II-dependent force patterning therefore emerges as a key regulator of cell–cell interactions.

## Results

### B cells exert pulsatile pulling forces on soft susbtrate

**Fig. 1:**
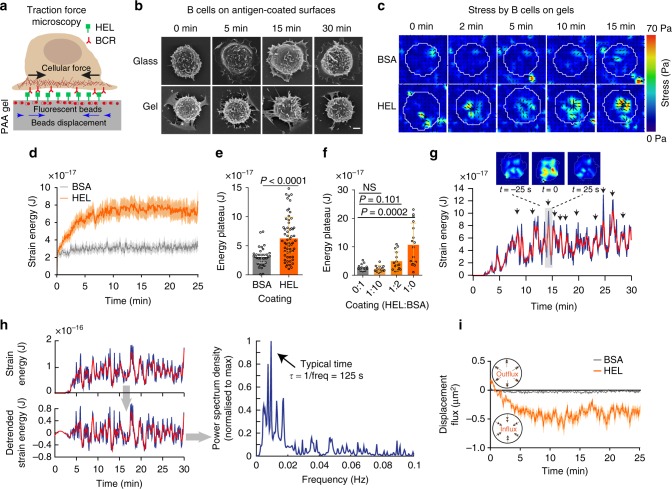
B cells show antigen-specific pulsatile traction forces on PAA gels. **a** Cartoon of traction force microscopy showing B-cell plated on antigen-coated polyacrylamide (PAA) gel containing fiducial markers. **b** Scanning electron microscopy of fixed B lymphocytes on HEL-coated glass and PAA gels, scale bar is 2 µm. **c** Time-lapse color maps of stress for HEL and control BSA condition; contractile stress can reach 70 Pa. **d** Comparison of average strain energy profile for HEL and BSA conditions, error bars represent mean ± SEM (*n* = 65 for HEL and *n* = 35 for BSA, five independent experiments, five mice), acquisitions were started before the arrivals of the cells to capture the initial time of contact and all cells were aligned at time zero. **e** Summary statistics of plateau of strain energy for HEL and BSA, error bar represents median ± IQR (*n* = 65 for HEL and *n* = 35 for BSA, five independent experiments, five mice), Mann–Whitney test was performed for statistical analysis. **f** Concentration-dependent increase in strain energy, error bars representing median ± IQR (*n* = 12, 13, 15, 16, 3 independent experiments, three mice), Mann–Whitney test was performed for statistical analysis. **g** Example of the strain energy curve for a single cell, plateau exhibit isolated peaks (see single stress maps). **h** Extraction of the typical pulsation frequency from the contractile energy: from the time series of a coordinated energy signal (smoothed in red), the signal is de-trended and the power spectrum density derived from it shows a maximum, hence a typical time scale for the pulsation. **i** Displacement flux showing the direction of displacement over time in HEL and BSA condition (mean ± SEM, *n* = 65 for HEL and *n* = 35 for BSA, five independent experiments, five mice). Source data are provided as a Source Data file

We used time-dependent traction force microscopy (TFM, see Methods)^13–15^ to analyse the spatio-temporal distribution of forces at the B-cell synapse (Fig. 1a). Primary naive B cells freshly purified from the spleen of mice expressing a hen egg lysozyme (HEL)-specific BCR were plated on gels coated with HEL or with bovine serum albumin (BSA) as a negative control. A rigidity of 500 Pa that matches the physiological rigidity of the macrophages that present the antigen to B cells in vivo was chosen^16^, as B cells were previously shown to behave differently when plated on gels of different rigidities^17,18^. Surprisingly, scanning electron microscopy (SEM) analysis showed no B-cell spreading on antigen-coated gels, spreading being observed on glass coated with equivalent amounts of antigen, as previously reported (Fig. 1b, see also Supplementary Fig. 1a). Instead, when B cells contacted antigen-coated gels, they exhibited pulsatile contractions (Supplementary Movie 1). To characterize this cell mechanical behavior, we quantified the stress (Fig. 1c) and the strain energy exerted on the substrate. We found that the strain energy displayed a growth phase lasting ~ 5 minutes followed by a plateau (Fig. 1d, e). This growth phase was barely observed in the absence of HEL (Fig. 1d, e, Supplementary Fig. 1b, Supplementary Movie 2) and the plateau displayed a clear antigen dose-dependence (Fig. 1f). Analyzing the single cell energy curve, we found that the plateau phase exhibited peaks in energy, corresponding to global cell contractions (Fig. 1g). Spectral analysis revealed a typical time-scale of 170 ± 10 s (median ± IQR) between each contractile event (Fig. 1h and Supplementary Fig. 1c). Measurements of the bead displacement field flux through the cell boundaries revealed that the forces detected were mostly directed inward (Fig. 1i). We conclude that, on substrates of physiological rigidity, B cells exert pulsatile forces directed towards the synapse center in an antigen-dependent manner.

### Force patterning at the immune synapse

**Fig. 2:**
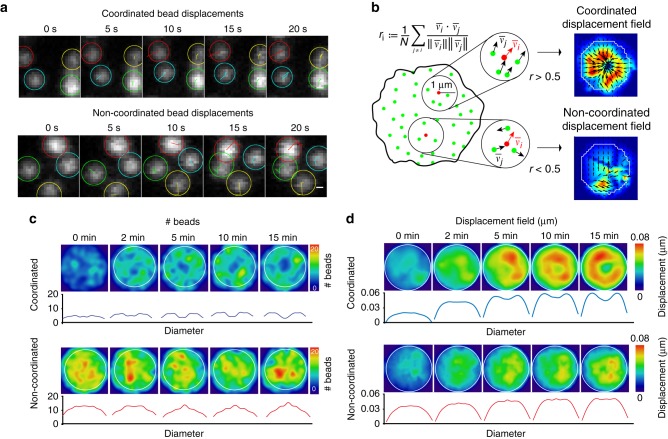
Forces at the synapse exhibit two components. **a** Time-lapse images of individual beads showing displacement tracks in coordinated and non-coordinated type of movements, scale bar is 0.3 µm. **b** Scheme showing the method used to separate the bead population in two pools, allowing extraction of coordinated and non-coordinated displacements. **c** Mean bead distribution in coordinated and non-coordinated pool of stress (*n* = 100 cells). Below: radial profile of the density map obtained by average spatial distribution of the beads by resizing all cells and interpolating each bead with a Gaussian kernel. **d** Mean displacement field map obtained by resizing and averaging (over all cells) at the indicated time point the individuals coordinated and non-coordinated stress maps

An important hypothesis used to build the algorithm for force calculation in typical TFM experiments is that the displacement of cell-associated beads is accompanied by the displacement of its neighbors. However, we consistently observed that certain beads did not display movements parallel to the ones of their neighbors (Fig. 2a). These apparently aberrant bead movements did not result from a modification of gel elasticity as the gel relaxed to its initial state upon cell detachment (Supplementary Fig. 2a). We hypothesized that they rather result from locally applied forces, which may be perpendicular to the synaptic plan. To test this possibility, we investigated the nature of these local displacements by splitting the pool of beads into two groups based on *r*, the correlation between the directions of displacement vectors with its neighbors in a range of 1 µm (Fig. 2b). For each frame, we classified the beads in two groups: coordinated (*r* *>* *0.5*) and non-coordinated (*r* *<* *0.5*) (see also Supplementary Note 1, Analysis of the coordinated and non-coordinated bead movements). Remarkably, these two types of movements were spatially segregated, as observed from average bead density maps and radial scans (Fig. 2c): the coordinated pool was located at the periphery of the synapse ( ~ 2–3 µm from the center), whereas the non-coordinated one was located at its center. Of note, the number of beads moving in a coordinated manner increased with time, reaching a plateau at ~ 3 min (Supplementary Fig. 2c), suggesting that force patterning occurs early upon cell–gel contact, most likely during the rising phase of the strain energy curve. A similar spatial segregation was observed when analyzing the displacement field calculated from each group of beads (Fig. 2d). Of note, because the TFM algorithm cannot be used for localized forces but only for tangential ones (Supplementary Note 1, Underestimation of the non-coordinated pool of forces), we could not compute the stress field in this analysis. We conclude that forces transmitted to the substrate present a specific spatio-temporal pattern at the immune synapse with a peripheral, centripetal, tangential pool opposed to a central, localized, and disorganized one.

### Tangential forces rely on myosin II-driven cell contraction

**Fig. 3:**
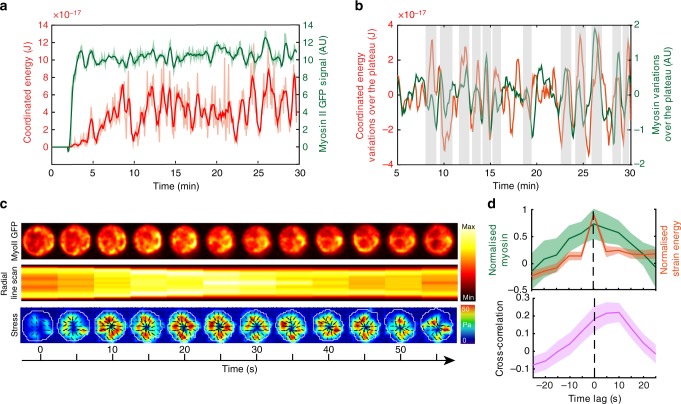
Myosin II recruitment is associated to global contractions. **a** Example of strain energy and myosin II GFP signal at the synapse. **b** The two signals (once de-trended) show concomitant peaks. **c** Time-lapse images showing dynamics of myosin II GFP, radial linescan (presented as a kymograph, time in *x* axis) and corresponding stress map: a contraction peak is visible at times *t* = 20–30 s. **d** Average of different peaks and cross-correlation between strain energy and myosin II peak intensity: myosin peak precedes the energy peak of ~ 5–10 s (Mean ± SEM, *n* = 40 peaks, from 15 cells, four independent experiments). Source data are provided as a Source Data file

We next investigated the role of the actomyosin cytoskeleton in force patterning. Monitoring myosin II-GFP dynamics showed that it displayed a pulsatile behavior similar to the one observed when analyzing the coordinated component of the cell contractile energy (Fig. 3a, b, Supplementary Movie 3). Zooming on a single energy peak showed that myosin II-GFP recruitment indeed coincided with maximal contractile stress (Fig. 3c). This was also visible when averaging the myosin II-GFP and energy signals over 40 different peaks (Fig. 3d). Cross-correlation analysis showed that myosin II-GFP peaks preceded the energy ones by few seconds, consistent with the motor being first recruited to the synapse and then triggering global contractions (Fig. 3d). These results strongly suggest that coordinated peripheral forces arise from global actomyosin contractions.

**Fig. 4:**
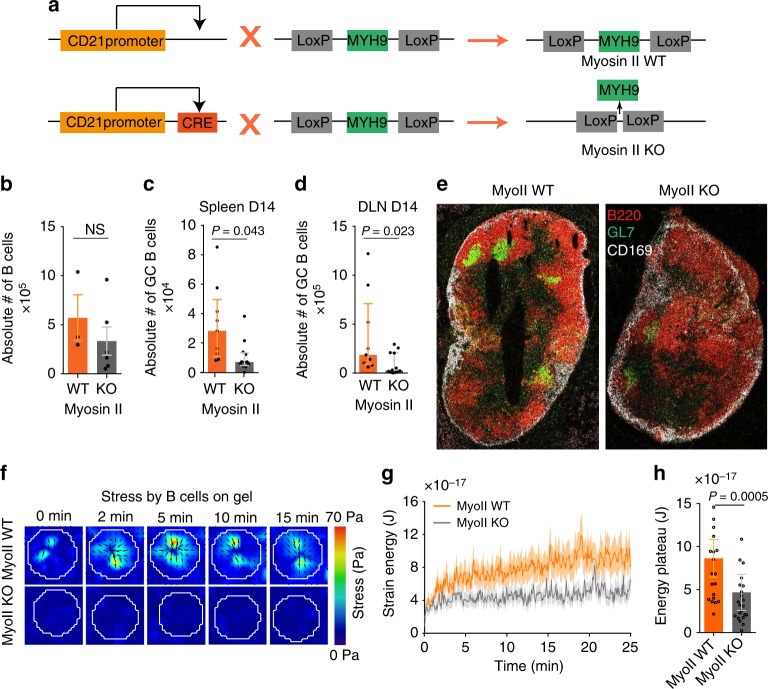
Myosin II is essential for force generation by B cells. **a** Genetic approach used to ablate Myosin IIA specifically in B cells: MyoII Flox mice are crossed with CRE + mice under CD21 promoter. **b** Absolute number of CD19-positive B cells in myosin II WT and KO mice inguinal lymph node (each dot represents one mice, two independent experiments, error bars represents mean ± SEM, Mann–Whitney test was performed for statistical analysis). **c** Absolute number of germinal center B cells in inguinal lymph node and **d** draining lymph node in myosin II WT and KO beads immunized mice (each dot represents one mouse, two independent experiments, error bars represent median ± IQR, Mann–Whitney test was performed for statistical analysis). **e** Histology image of draining lymph node from immunized mice showing B cells (B220), germinal centers (GL7), and sub-capsular sinus macrophages (CD169); images highlight scattered germinal center B cells in myosin II KO mice. **f** Time-lapse images of stress color maps for myosin II KO and WT conditions, forces are almost absent in myosin II KO cells. **g** Average energy profile for myosin II KO and WT conditions, error bars represent Mean ± SEM (*n* = 22, 23, four independent experiments, four mice). **h** Summary statistics of plateau of strain energy for myosin II KO and WT, error bars represent median ± IQR (*n* = 22, 23, four independent experiments, four mice), Mann–Whitney test was performed for statistical analysis. Source data are provided as a Source Data file

To assess the involvement of myosin II in force generation, we used conditional knockout mice in which *MYH9*, the gene coding for the main myosin II isoform expressed in lymphocytes (Immunological Genome Project, http://www.immgen.org), was deleted in B cells using the *CD21-cre* transgene (Fig. 4a, Supplementary Fig. 3a). No difference in the number of B cells in lymph nodes was observed between WT and myosin II KO mice (Fig. 4b). However, germinal centers were disorganized and reduced in number in the spleen and lymph nodes of immunized myosin II KO mice (Fig. 4c–e and Supplementary Fig. 3b). Thus, myosin II is required for B-cell responses in vivo, which is consistent with recently published results^19^, validating our experimental model. Remarkably, monitoring of the forces exerted on HEL-coated gels showed that the contractile strain energy of most myosin II-deficient B cells was considerably decreased (Fig. 4f–h, Supplementary Movie 4). Similar results were obtained when inhibiting myosin II with para-nitro-blebbistatin (Supplementary Fig. 3c). SEM analysis showed that myosin II KO spleen B cells did not show major morphological differences as compared with their wild-type counterpart (Supplementary Fig. 3d). We conclude that tangential forces exerted at the B-cell synapse are mediated by myosin II-driven centripetal cell contraction.

### Localized forces result from protrusive actin patches

**Fig. 5:**
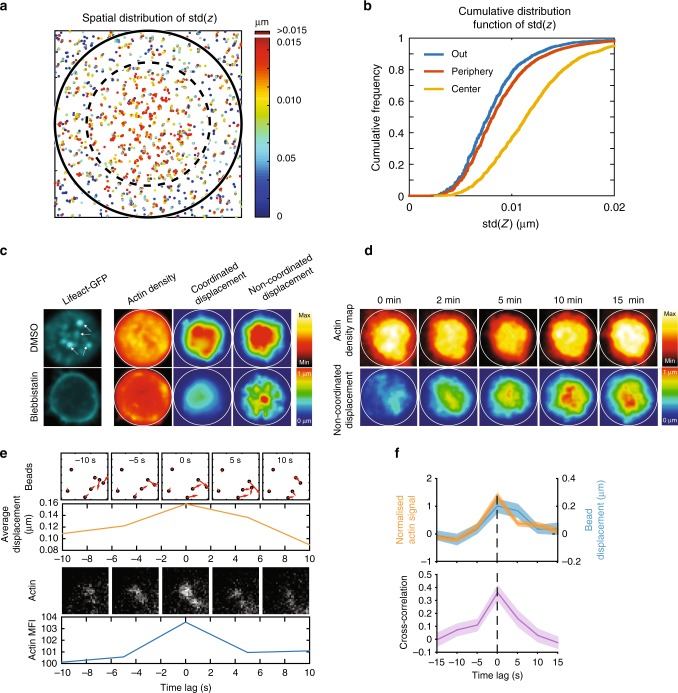
Actin dynamics at the synapse. **a** Space distribution of bead displacement in the z direction: each point represents the standard deviation of the *z* position of a single bead on a 10 frame sequence; all beads have been projected on a size and aspect ratio normalized cell (inner circle at 2/3 of the cell diameter) represents the central portion of the synapse: the figure show that the displacement in *z* is higher in the center of the synapse (8422 points, superposition of 14 cells, one representative experiment). **b** Cumulative distribution for the *z* displacement color-coded in figure **a**, the central fraction of beads shows significantly higher displacement in *z* (*P* < 0.0001 for all comparison, Kolmogorov–Smirnov test). **c** Left, single cell showing actin patches in the center of the cell which are lost in case of para-Nitroblebbistatin treated cells. Right, mean actin distribution, mean coordinated and mean non-coordinated force density color map in control and para-Nitroblebbistatin treated B cells (*n* = 12). **d** Actin distribution over time correlates with non-coordinated force distribution (images are average projection of 12 cells over all time points). **e** Time-lapse images showing simultaneous appearance of an actin patch and non-coordinated bead displacements, arrowhead showing direction and magnitude of displacement; graphs below the images represent the respective signals integrated over a square of 2 µm × 2 µm. **f** Average of the signals quantified in **e** and average cross-correlation: both signals appear simultaneously and show a peak simultaneously with no lag (mean ± SEM, *n* = 89, from nine cells, two independent experiments). Source data are provided as a Source Data file

**Fig. 6:**
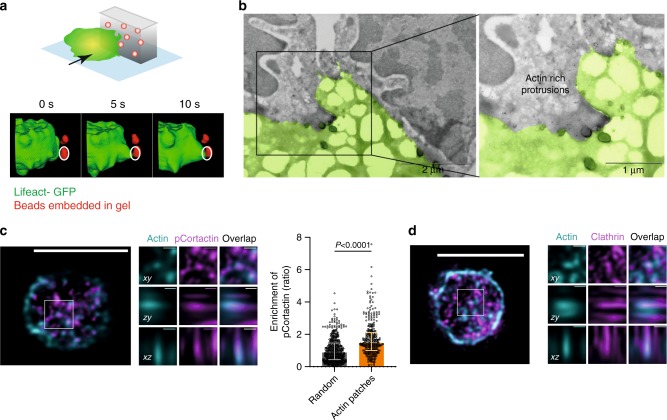
Characterization of actin patches. **a** Protrusions associated with bead movement (red) on antigen-coated gel pieces presented laterally to B cell (green), 3D reconstruction. **b** Electron microscopy image showing actin-rich protrusions (marked by gold beads) through PAA gel (scale bar 0.2 µm). **c** Immunostaining of actin (cyan) and phospho-cortactin (magenta) and zoomed orthogonal projections on different planes (scale bar 5 µm, zoom 0.5 µm); images show colocalization (quantify on the right as ratio of magenta signal in the cyan region, control is the enrichment of the signal in random position, Median ± IQR, 26 cells, two independent experiments, two mice, Mann–Whitney test). **d** Immunostaining of actin (cyan) and clathrin (magenta) and zoomed orthogonal projections on different planes (scale bar 5 µm, zoom 0.5 µm); images show partial colocalization (not quantified, scale bar 5 µm, zoom 0.5 µm). Source data are provided as a Source Data file

We next investigated the nature of the non-coordinated force component. By analyzing the *Z* displacements of each bead (quantified in the standard deviation of the position over 60 s), we observed that their movement in *Z* was indeed higher at the synapse center as compared with the periphery (Fig. 5a, b). This finding suggested that non-coordinated forces might result from local 3D movements of the cell. Strikingly, analysis of LifeAct-GFP dynamics at the cell–gel interface showed the presence of actin patches at the center of the synapse (Fig. 5c, d and Supplementary Movie 5), where most of bead movements in *Z* were detected (Fig. 5a). Accordingly, we found that actin patches and non-coordinated bead displacements were correlated in space and time (Fig. 5e, f). This result indicates that actin patches might be responsible for localized non-coordinated bead movements, suggesting that they correspond to protrusive structures. Consistent with this hypothesis, when presenting laterally pieces of antigen-coated gels to LifeAct-GFP B cells, we observed actin-rich protrusions that penetrated within the gel and were associated to bead movement (Fig. 6a). This experiment was motivated by the fact that the presence of the gel strongly limits imaging resolution in *Z*, compromising the analysis of these protrusive structures in B cells plated on 2D antigen-coated gels. However, we could confirm the existence of actin-rich protrusions in these cells by cryo-electron microscopy (Fig. 6b and Supplementary Fig. 4). Actin patches colocalized with phosphorylated Cortactin, a hallmark of invadosome-like protrusions previously observed in other cell types including T cells^20,21^ (Fig. 6c) and, partially, with clathrin (Fig. 6d), suggesting that clathrin-mediated endocytosis might locally take place. Other podosomes hallmarks (vinculin, phosphorylated paxillin, and fascin) were not found to colocalize with actin (Supplementary Figs. 5a–c). We conclude that non-coordinated forces localized at the center of the immune synapse most likely result from the formation of protrusive actin patches that resemble invadosome-like protrusions.

### Antigen extraction occurs in actin protrusive patches

**Fig. 7:**
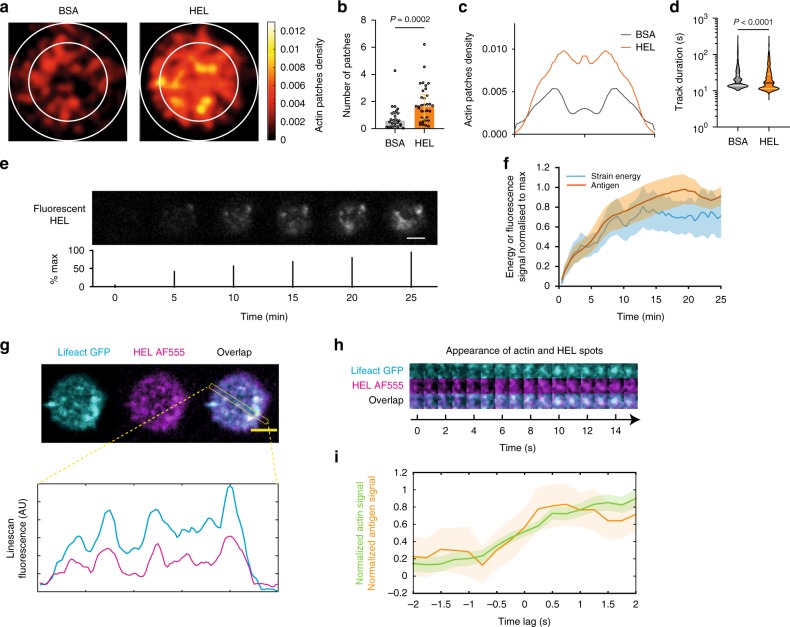
Actin patches correlate with antigen Internalization. **a** Average distribution of actin patches (obtained by tracking and convoluting the results with a Gaussian kernel): internal circle (2/3 cell diameter) corresponds to the central portion of the cell (scale bar represents the integrated density as number/cell/5 min). The distribution in HEL-coated gel is very different compared with the BSA coated gel in **b** number of beads (Mann–Withney test, Median ± IQR) and **c** radial distribution (BSA: 25 cells, HEL: 34 cells, three different mice). **d** Effective diffusion coefficient of actin patches (violin plot with Median ± IQR, *n* = 301 trajectories (34 cells) for HEL and *n* = 84 trajectories (25 cells) for BSA, Mann–Whitney test). **e** Time lapse of the extraction of fluorescent HEL (below: percentage of the maximum, scale bar 3 µm). **f** Strain energy and antigen gathering (Mean ± SEM, *n* = 15, five independent experiments); signals are normalized to the maximum to highlight similarity in the kinetics. **g** Example of colocalization of the actin patches (lifeact-GFP) and fluorescent HEL (AF555), bottom linescan on the colocalization panel (scale bar 3 µm). **h** Zoom on an actin (and HEL) patch showing the concomitant arrival of the two signals. **i** Quantification of the signal in **g** measured over a square of 1 µm **×**  1 µm: both signals appear simultaneously (mean ± SEM, *n* = 21, from six cells). Source data are provided as a Source Data file

We next investigated the dynamics of these actin patches in the presence or absence of antigen. We found that only few actin patches formed on BSA, which does not engage the BCR (Fig. 7a, b). In addition, actin patches were more peripheral in absence of BCR stimulation, compared with their central localization in presence of HEL (Fig. 7c). Patch tracking further showed that HEL increased their lifetime (Fig. 7d). Altogether, these data suggest that the presence of BCR-specific antigens facilitate the stable formation of actin patches that protrude into the gel and are localized at the center of the synapse.

These results prompted us to investigate whether antigen extraction occurs at these protrusive actin patches. For this, we recorded B cells plated on gels coated with fluorescently labeled HEL. Surprisingly, we observed that fluorescence was quenched when HEL was linked to the gel, being only detected upon HEL detachment (Supplementary Fig. 6a). This unexpected observation provided us with a robust system to monitor HEL extraction together with force generation or formation of actin patches. We observed a gradual antigen detachment starting as soon as B cells contact the gel surface and slowing down ~ 5 minutes later (Fig. 7e, f and Supplementary Movie 6). This crossover time corresponded to the time at which the plateau was reached in the energy curve. Strikingly, the appearance of actin patches at the center of the synapse coincided in space and time with the appearance of HEL clusters (Fig. 7g, h and Supplementary Movie 7). However, when coating the gel with both specific (HEL) and non-specific (Ovalbumin) fluorescent antigens, we observed that only HEL was extracted (Supplementary Fig. 6b). This result implies that antigen extraction does not only rely on the formation of actin patches but further requires specific antigen binding to the BCR. Altogether, our findings strongly support a model where actin protrusions that form at the center of the immune synapse allow the local extraction of BCR-associated antigens.

### Actin patch formation and antigen extraction rely on myosin II

**Fig. 8:**
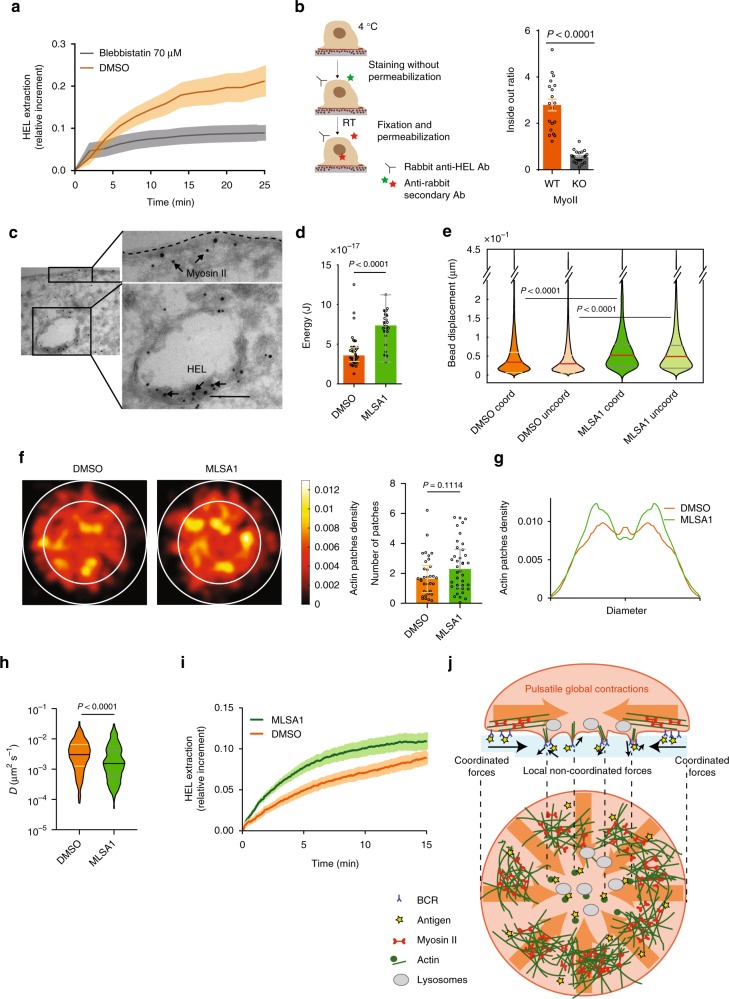
Antigen is internalized through a myosin II-dependent mechanism. **a** Antigen gathering over time in control and para-Nitroblebbistatin treated cells (error bars show mean ± SEM, *n* = 15 DMSO, *n* = 9 Blebbistatin). **b** Scheme of the inside–out experiment and relative quantification: ratio of internalized (inside) and not internalized (outside) antigen in myosin II KO and WT B cells (error bars show mean ± SEM, *n* = 29 Myosin II WT, *n* = 21, Myosin II KO, three independent experiments, *t* test). **c** Scanning electron microscopy images showing internalized antigens and its proximity to myosin II (scale bar 0.2 µm). **d** Statistics of plateau of strain energy for MLSA1-treated (MLSA1) and untreated (DMSO) cells (error bar show median ± IQR, *n* = 41 for DMSO and *n* = 30 for MLSA1, three independent experiments, three mice, Mann–Whitney test): contractile energy is strongly increased in the treated cells. **e** Statistics of the bead displacement in the coordinated and uncoordinated compartment for treated and untreated cells: both coordinated and uncoordinated movements are significantly increased in treated cells (in red median ± IQR, Mann–Whitney test, *n* = 41 for DMSO and *n* = 30 for MLSA1, three independent experiments, three mice, total of > 4400 beads). **f** Average distribution of actin patches (obtained by tracking and convoluting the results with a Gaussian kernel): internal circle (2/3 cell diameter) corresponds to the central portion of the cell (scale bar represents the integrated density as number/cell/5 min). The distribution in MLSA1-treated cells is different compared with the untreated (DMSO) cells neither in number of beads (it increases but not significantly) nor **g** in radial profile (Mann–Withney test, Median ± IQR, *N* = 34 DMSO, *N* = 38 MLSA1, three independent experiments, three mice). **h** Apparent diffusion coefficient of actin patches: MLSA1-treated cells exhibit less mobile patches (number of tracks: *N* DMSO=301, *N* MLSA1=492, Mann–Whitney test). **i** Antigen extraction profile for MLSA1-treated and untreated cells: treated cells extract antigen faster than untreated (*N* = 55 DMSO, *N* = 53 MLSA1, three independent experiments, three mice). **j** Model: myosin II-driven global peripheral contractions (shear coordinated forces) allow the endocytic machinery to build up in the center, where antigen is extracted through actin protrusions associated to the generation of localized forces. Source data are provided as a Source Data file

So far, our data show that myosin II-mediated pulsatile contractions account for tangential coordinated forces generated at the synapse periphery, whereas actin protrusions are responsible for localized 3D forces at the synapse center as well as for antigen extraction. To assess whether these two spatially segregated functions of the actomyosin cytoskeleton are or not linked, we analyzed the impact of myosin II inhibition on actin patch formation. We found that Blebbistatin treatment strongly decreased the formation of actin patches and reduced the non-coordinated bead displacements localized at the synapse center (Fig. 5d). Consistently, myosin II inhibition almost cancelled the extraction of gel-associated HEL (Fig. 8a). Thus, myosin II is needed for actin patch formation and antigen extraction at the synapse center. Of note, antigens were not only detached from the substrate, but also internalized within B cells as shown by inside–out HEL staining (Fig. 8b). In agreement with these findings, myosin II was detected by cryoimmuno-electron microscopy at the cytosolic face of vesicles containing internalized HEL (Fig. 8c). In contrast, HEL was mainly found at the cell surface in myosin II KO B cells (Supplementary Fig. 6c).

Intriguingly, unlike actin-, myosin II-containing patches were not observed at the synapse center. This points to an indirect role of myosin II in patch formation rather than a direct one. We therefore hypothesized that myosin II-mediated contractions might facilitate the formation of the central actin patches for antigen extraction. To test this hypothesis, we evaluated the impact of myosin II-contractility stimulation on actin patch formation and antigen extraction. For this, we used MLSA1, an agonist of the lysosomal calcium channel TRPML1, which locally enhances myosin II flows and activity in dendritic cells^22^. We found that MLSA1-treated B cells showed increased contractile energy (Fig. 8d), both non-coordinated and coordinated displacement fields being enhanced (Fig. 8e). Noticeably, although MLSA1 treatment had a minor effect on the number of actin patches (Fig. 8f) and their distribution (Fig. 8g), it strongly decreased their diffusion coefficient (Fig. 8h), indicating that patches became more stable when myosin II contractility was enhanced. Consistent with this result, antigen extraction was significantly faster and more efficient in MLSA1-treated B cells (Fig. 8i). Thus, although inhibition of myosin II abolishes force generation, actin patch formation and antigen extraction, stimulation of its activity enhances these three events, strongly suggesting that they are most likely functionally linked.

To address the existence of this functional link, we built a theoretical model (Fig. 8j). The model considers that molecules associated to the membrane can both diffuse and be advected by an intermittent flow induced by actomyosin contractions. At the cellular scale, the diffusion time of these molecules (the time necessary for molecular patterns such as actin patches to disappear) is more than 5 min, i.e., it is longer than the typical period of the 2–3 min. pulsations detected (see Supplementary Note 1, myosin II-driven pulsatile contractions can lead to central patterns). Noticeably, calculations show that this condition is sufficient to generate a radial gradient of advected molecular components. This analytical argument therefore supports the idea that global myosin II pulsatile contractions promote the centripetal transport/accumulation of molecules that in turn facilitate the formation of stable protrusive actin patches where antigen extraction occurs. This spatial organization of the actomyosin cytoskeleton leads to force patterning at the immune synapse, with pulsatile tangential (2D) peripheral forces resulting from global myosin II contraction and 3D disorganized central forces being produced by local actin protrusions.

## Discussion

We here show that forces are patterned at the immune synapse of B lymphocytes. Consistent with others’ findings^23^, we observe the build up of a contractile concentric ring upon BCR activation, but in addition to this, we detect localized forces mainly located at the center of the synapse. We propose that force patterning results from centripetal pulsatile actomyosin contractions that lead to the segregation of molecular components at the cell-antigen interface. This scenario is distinct from the one described at the immune synapse formed by lymphocytes interacting with antigen-functionalized lipid bilayers, as molecular segregation is driven by centripetal actin flow in these cells^10,24,25^, which were not detected in B lymphocytes interacting with antigen-coated gels. This might result from the fact that (1) on gels, cells are anchored to the substrate, allowing force transmission, which is not the case in fluid lipid bilayers, and/or (2) gels are several orders of magnitude softer than glass surfaces.

We observed protrusive actin patches that form at the synapse center, where antigen extraction occurs, and which resemble invadosome-like protrusions. This is consistent with in vitro studies showing that when an actomyosin active gel is coupled to the cell membrane, it can indeed form actin patches as long as a sufficient number of contractile elements are present^26,27^. It is therefore likely that shear coordinated forces generated by myosin II pulsatile contractions at the synapse periphery do not directly contribute to antigen extraction but rather help the cell building stable protrusive actin structures where extraction occurs (see Supplementary Note 1, force required for antigen extraction). It therefore appears that both global contractility and local force generation are involved in antigen extraction, reconciling the two models previously proposed. Interestingly, the growth-plateau regimes that we observed are reminiscent of the spreading-contraction phases observed on fluid substrates.

We found that the lifetime of protrusive actin patches increases in the presence of BCR-specific antigens. This suggests that similarly to T lymphocytes, B cells might probe their environment through unstable actin protrusions, which are then stabilized upon antigen binding to the BCR^28^. Antigen internalization could occur by endocytosis at the tip of protrusions as described for both clathrin-mediated endocytosis^29^ and clathrin-independent IL2 receptor endocytosis^30^. Interestingly, actin-rich pod-like structures have been recently described as sites of antigen internalization in human Light Zone B cells plated on activating plasma membrane sheets^12^. Although it is not clear at this stage whether these actin pods are the protrusive structures we here describe, our data provide a putative mechanism for their formation.

Our results show that, in the presence of antigen, actin patches form at the center of the synapse, in agreement with previous studies showing that this is indeed a privileged site for antigen internalization. Actin-rich endocytic structures might preferentially form at the synapse center owing to a local drop in membrane tension, as recently described during phagocytosis in macrophages^31–33^. In this context, pulsatile peripheral actomyosin ring could contribute creating a gradient of lipid and therefore of tension (even without need for a proper diffusion barrier) with a reduced membrane tension at the center of the synapse. Alternatively, the actomyosin peripheral ring might act as a mechanical dumper by sealing the synapse and isolate its center from external mechanical noise, for example, owing to lymph node/vessel contractions or cell movements/proliferation. This could improve antigen affinity mechanical discrimination by the BCR^8^ (see Supplementary Note 1, affinity discrimination and energy scales).

We observed that lysosomal calcium release enhances myosin II-driven peripheral forces as well as actin patch formation and subsequent antigen extraction. This is particularly appealing as lysosomes have been shown to be recruited upon centrosome polarization to the B-cell immune synapse^9,34^, which would then be locally available for calcium release. We do not exclude that local release of calcium might also promote the activity of other myosin motors such as class I myosins, which are typically required for clathrin-mediated endocytosis^35–37^. This would help coupling global myosin II contractions at the cell periphery with local endocytosis at actin patches. Indeed, although the minimal force to activate the BCR is 16 pN (as measured by DNA tension sensors^38^), the stall force of a single myosin motor is <2 pN^39^, indicating that the action of at least eight myosin motors is required to activate the receptor. Moreover, higher forces have been reported in antigen extraction: 56 pN rupture forces have been measured^38^ as well as biotin–streptavidin bond ruptures^8^ (requiring 160 pN or 80 motors). Single contractile elements made of tens of motors can achieve large force peak (recent experiments report forces up to 1 nN^40^), provided that the activity of several motors is properly coordinated in order to achieve efficient antigen extraction, which could be orchestrated by local lysosomal calcium release. This further implies that lysosomal polarization to the immune synapse might be needed to stimulate mechanical extraction of surface-tethered antigens, in addition to their known role in antigen proteolysis and processing^41^.

Spatio-temporal force patterning was first highlighted in the context of tissues^42^, cell adhesion to substrate^43–45^, and cell motility^46^. Our study shows that it might be a more general and basic feature of cell–cell interfaces where the engagement of surface receptors leads to both juxtacrine signaling and ligand endocytosis. We found that myosin II intervenes in this process as a global master organizer of forces and actin organization, and thus as an indirect but key actor of endocytosis, which is essential for adaptive immunity. We anticipate that this study should set the ground for future work aimed at exploring force patterning in additional cell-to-cell communication models.

## Methods

### Mice and cells

Mice with a conditional deletion of myosin II in B cells were generated by backcrossing mice carrying a floxed myosin II allele *(MyosinII flox/flox)*^47^ with mice expressing the Cre recombinase under the control of the CD21 promoter *(CD21-cre*^*+/−*^*)*. Mice expressing the HEL-specific MD4 receptor were also crossed with mice carrying a floxed myosin II allele. Mice were crossed at an age of 8–10 weeks, and Cre^−^ littermates were used as WT controls. The transgenic MD4, Lifeact-GFP and myosin II-GFP mouse lines have been described elsewhere^48,49^. This resulted in all the desired genetic combinations being obtained in the C57BL/B6 background, and the corresponding breeding controls were systematically used. The experiments were performed on 8–10-week-old male or female mice. Animal care conformed strictly to European and French national regulations for the protection of vertebrate animals used for experimental and other scientific purposes (Directive 2010/63; French Decree 2013-118). Immunization experiments carried out at the Karolinska Institute were performed according to local ethical committee guidelines (N11/13). Mature spleen B cells were purified with the MACS kit (130-090-862). B cells were cultured^9^ in Rosewell Park Memorial Institute (RPMI) 1640—GlutaMax-I supplemented with 10% fetal calf serum, 1% penicillin–streptomycin, 0.1% b-mercaptoethanol and 2% sodium pyruvate.

### Antibodies and reagents

The following reagents were used: 100 µg/ml HEL (Sigma), 100 µg/ml BSA (Euromedex), 40% polyacrylamide (PAA, Biorad), 2% bis-poylacrylamide (Biorad), 3-aminopropyltrimethoxysilane (Sigma), 0.2 µm Alexa647 Fluospheres (Thermo Fisher, F8807), Sigmacote (Sigma), ammonium persulfate (Sigma), TEMED (ICN Biomedicals), Sulfo-SANPAH (Thermo Fisher), and the Alexa555 protein labeling kit (A30007, Molecular Probes). The following primary antibodies were used: rabbit anti-HEL (Abcam,1/100), human anti-GFP (Institut Curie, 1/200), rabbit Anti-phospho-Cortactin (pTyr^466^) (SAB4504373, Sigma-Aldrich, 1:200), rabbit anti Clathrin (Cell Signalling 4796, 1:50), Alexa Fluor 488 Phalloidin (A12379 Invitrogen 1:200), Alexa Fluor 647-conjugated anti-phalloidin (Thermo Fisher, 1/200), anti-myosin IIA heavy chain (Covance, 1/500). The following secondary antibodies were used: Alexa Fluor 488-conjugated goat anti-rabbit IgG (Life Technologies, 1/200), Alexa Fluor 488-conjugated goat anti-human IgG (Life Technologies,1/200), Alexa Fluor 405 Goat anti-Rabbit IgG (H + L) (A31556 Thermofisher 1:200), Alexa Fluor 546 Goat anti-Mouse IgG1 (A21123 Thermofisher 1:200).

### Inhibitors and drugs

Blebbistatin: cells were incubated with 70 µm para-nitro blebbistatin (Optopharma) for 40 minutes at 37 °C in RPMI media before the experiments, unless otherwise stated. MLSA1: cells were incubated with 10 µm mucolipin synthetic agonist 1 (MLSA1, TOCRIS) for 30 minutes at 37 °C in RPMI media before the experiments. Dimethyl sulfoxide (DMSO) was used as a control.

### Live cell traction force microscopy

Fluorodishes containing gels were placed under a microscope focused on the plane of beads. In total, 1 × 10^5^ B cells were added to the plate (time 0), and images were acquired over time. Images were acquired at 37 °C, under an atmosphere containing 5% CO_2_, with an inverted spinning disk confocal microscope (Eclipse Ti Nikon/Roper spinning head) equipped with a × 60 (1.4 numerical aperture) oil immersion objective and a CoolSNAP HQ2 camera (pixel size 6.4 µm) with MetaMorph software (Molecular Device, France); time lapse were typically 1image/5 s and last minimum 15 mins.

### Preparation of PAA gel substrates

PAA gels were produced in 35-mm FD35 fluorodishes (World Precision Instruments, Inc). These dishes were first treated by UV irradiation for 2 minutes, and then with 3-aminopropyltrimethoxysilane for 5 minutes. The dishes were washed thoroughly in distilled water and dried before preparation of the PAA gels. Hydrophobic 12mm diameter coverslips were prepared by incubation in Sigmacote for 3 minutes, followed by thorough washing and drying. A 500 Pa gel was prepared by diluting 40% PAA and 2% bis-acrylamide solutions to obtain stock solutions of 12% acrylamide/0.1% bis-acrylamide. We sonicated 167 μl of this solution with 1% of 0.2 μm carboxilated fluorescent (660/680) beads (ThermoFisher Scientific), and then added 1.67 µl of the 10% ammonium persulfate (APS) stock solution and 0.2µL of TEMED and mixed thoroughly, to initiate polymerization. A volume of 9 μl of the PAA mixture was immediately pipetted onto the surface of the Fluorodish and a Sigmacote (Sigma-Aldrich) activated coverslip was carefully placed on top to sqeeze the gel to a thickness of about 80µm. Fluorodishes were immediately inverted, to bring the beads to the surface of the gel. Polymerization was completed in 45 min at room temperature and the top coverslip was then slowly peeled off and immediately immersed in phosphate-buffered saline (PBS). Sulfo-SANPAH (Sigma-Aldrich), a surface functionalizing reagent with an amine-binding group and a photoactivable azide group, was used to crosslink molecules to the surface of the gel. Sulpho-SANPAH (150 μl of 0.5 mg/ml stock) was attached to the gel surface through UV light activation for 2 minutes followed by 2x PBS washing (procedure repeated 2 times). Gels were washed thoroughly with PBS 3 times and finally coated with 100 µl (100 μg/ml) HEL or BSA, by overnight incubation at 4 °C. Gels were washed thoroughly with PBS 3 times and pre-incubated with medium at room temperature at least 30 min before experiments.

### Characterization of the PAA gels

The Young’s modulus of PAA gel was measured by bead indentation and calculated using a Hertz model for an elastic substrate with finite thickness^50^. Glass beads of 0.25 mm radius were deposed on the gel and their indentation was measured using confocal stacks. Gel height was determined by focussing on the bottom and top of the gel. The force inserted in the Hertz formula was computed theoretically as the weight of the glass bead (density = 2.2 kg/m^3^ and radius 0.25 mm) minus the buoyancy in water. PAA gels in our system remains in the range of 400–650 Pa.

### Quantification of amount of antigen on PAA gel and glass

We ensured that the difference of spreading on gel and on glass was not owing to the amount of antigen-coated on different substrates (it is harder to coat gels with protein due to the inherent hydrophobicity of the PAA). We inferred the amount of antigen required for coating the glass with an equivalent concentration of antigen on gel (100 µg/ml) by taking images at different concentration on glass and comparing with the fluorescent intensity obtained on the gel of 100 µg/ml. Respective glasses and gels were coated with HEL overnight at 4 °C and later stained by using rabbit anti-HEL primary antibody at 37 °C, eventually staining with anti-rabbit alexa-488 secondary antibody. Images were acquired using laser scanning microscope (Leica) with a × 40 1.4 NA oil immersion objective with 5% 488 laser. Mean fluorescence intensity at different point follows a logarithmic curve that suggests the equivalent concentration on glass is 0.14 µg/ml.

### Immunofluorescence

B cells plated for 30 min on PAA gels were fixed by incubation with PFA for 15 min at RT, washed 3× with PBS, permeabilized 5 min with Triton0.1%, washed 3 × with PBS. The sample was blocked for 30 min with CLSM buffer (PBS, 20 mm Glycine, 3% BSA), washed, blocked 10 min with Mouse Fc block (1:100 in CLSM buffer), washed, then incubated overnight at 4 degrees with primary antibodies (anti-phospho-Cortactin and anti-Clathrin) diluted in CLSM buffer. Secondary antibodies were incubated 1 h at RT. Samples were mounted so that cells could be imaged without going through the PAA gel. Samples were imaged using a laser scanning microscope (Leica) with a × 60, NA 1.3 oil immersion objective. Images were deconvoluted using Huygens software. Enrichment of p-Cortactin is quantified with custom-made ImageJ macro. Actin patches were detected average intensity of p-Cortactin in a disk around this patch was measured and then divided by the average intensity of p-Cortactin in the cell. This was compared with the same analysis, but taking random points in the cell instead of actin patches detection.

### Inside–out immunofluorescence

B cells were plated on PAA gels and incubated for 15 min at 37 °C. The cells were then transferred to 4 °C and Fc receptors were blocked for 10 min using Fc blocker (BD, 1/200). The cells were washed with PBS and incubated with rabbit anti-HEL antibody at 4 °C for one hour and then with Alexa Fluor 488-conjugated anti-rabbit IgG secondary antibody for one hour. The cells were moved to room temperature, fixed by incubation with 4% PFA for 10 minutes and permeablized by incubation with PBS plus 0.2% BSA and 0.05% saponin. The cells were then incubated with rabbit anti-HEL antibodies for 1 hour, washed with PBS–BSA-SAPONIN, and incubated with the Alexa 546-conjugated anti-rabbit IgG secondary antibody for one hour at room temperature. The cells were washed several times in PBS and then used for imaging. Images were acquired using laser scanning microscope (Leica) with a × 40 1.4 NA oil immersion objective.

### SEM imaging

Cells were fixed overnight at 4 °C in 2.5% glutaraldehyde in phosphate buffer on 0.2 µg/ml HEL coated on glass and 100 µg/ml HEL coated on PAA gel. They were dehydrated in a graded series of ethanol solutions, then dried by the CO_2_ critical-point method, with an EM CPD300 (Leica microsystems). Samples were mounted on an aluminum stub with silver lacquer and sputter-coated with a 5 nm layer of platinum, with an EM ACE600 (Leica Microsystems). Images were acquired with a GeminiSEM 500 (Zeiss).

### Electron microscopy

Immunoelectron microscopy was performed by the Tokuyasu method (Slot & Geuze, 2007). Double-immunogold labeling was performed on ultrathin cryosections with protein A-gold conjugates (PAG) (Utrecht University, The Netherlands). Electron micrographs were acquired on a Tecnai Spirit electron microscope (FEI, Eindhoven, The Netherlands) equipped with a Quemesa (SIS) 4k CCD camera (EMSIS GmbH, Münster, Germany).

### Western blotting

B cells were lysed at 4 °C in lysis buffer (10 mm Tris HCL pH 7.4, 150 mm NaCl, 0.5% NP40). Cell lysates were loaded onto mini-PROTEAN TGX sodium dodecyl sulfate polyacrylamide gel electrophoresis gels, which were run at 200 volts and 65 mA. The bands on the gel were transferred onto polyvinylidene fluoride membranes (Trans-Blot Turbo Transfer). Membranes were blocked with 5% BSA in 1 × tris-buffered saline (TBS) (Tris-buffered saline)–0.05% Tween-20 and incubated overnight at 4 °C with primary antibodies and then for 60 min with secondary antibodies. Western blots were developed with Clarity Western ECL substrate, and chemiluminescence was detected with a ChemiDoc imager (all from BioRad).

### Density map analysis

On movie reconstruction, individual cells were cropped with ImageJ software. For signal mapping, the images obtained for each individual cell were aligned in a single column. Cell size normalization was applied to each time point, based on mean cell size, with background subtraction. We obtained a mean behavior for each cell, by projecting every time point onto the average. The mean behavior of the population was then determined, by projecting the mean signal of every individual cell at a given time point. This procedure was performed with a custom-designed ImageJ-compatible macro. A similar procedure was used to map stresses, except that the real stress value was used, without normalization. For bead density analysis, we smoothed positions with a two-dimensional Gaussian kernel of radius 3 pixels to obtain a density map, as described by Schauer and coworkers^51^. These last two analyses were performed in Matlab. Similar analysis was carried out for the actin patches density (despite the normalization that was done per cell and on a time windows of 5 minutes, to pool observations done with different frame rates).

### Myosin and energy peak analysis

Maxima of the coordinated energy were isolated manually and a sequence of 11 frames around each maximum isolated and aligned to the maximum. The average Myosin II-GFP fluorescence was integrated in the area of the cell and aligned blindly following the energy sequence alignment. The pieces of signals were offset to zero and normalised to the maximum, averaged and plotted. For the correlation analysis the signals were cross-correlated and the average cross-correlation plotted.

### Actin patch and displacement analysis

Actin patches were isolated manually and the signal integrated in a square of 2 × 2 µm. In time sequence of 11 frames were considered separately. The signal of the displacement was computed as average absolute length of the displacement vector of the non-coordinated beads population in the same square used for the actin signal. Each sequence of actin signal was offset to zeros and aligned according to the maximum in fluorescence. The displacement signal was aligned blindly following actin ones. The pieces of signals were averaged and plotted. For the correlation analysis the signals were cross-correlated and the average cross-correlation plotted.

### Actin and HEL patches analysis

To show the simultaneous appearance of HEL and actin patches we performed an analysis similar to the one described above (but at high acquisition rate, 2 fps), in a smaller sequence (11 frames) and in a smaller window (1µm × 1µm) were performed. The only difference is that in this case the HEL signal was aligned first to the point of appearance and the actin signal was blindly translated.

### Actin patches tracking

B cells from Lifeact-GFP MD4 mice were settled onto a PAA gel coated with either BSA or HEL. Cells were allowed to settle for 10 min before imaging with an inverted spinning disk confocal microscope (Eclipe Ti Nikon/Roper Spinning head) equipped with a × 40 Water immersion objective 1.4 NA and a CoolSNAP HQ2 camera (pixel size 6.4  μm) with Metamorph software (Molecular Device, France). Time lapse were typically 1 image/6 s, taking 10 images stack with dz = 0.4 μm, and last 6 min. The acquisition were bleach-corrected and projected in *z*, before cropping the cells. Patches were tracked, excluding the ones on the cortex, using ImageJ (TrackMate). Tracks were further analyzed on Matlab to extract the diffusion coefficient D (on tracks of length *n* frames (*n>3) *it was obtained as a linear fit without offset of the first *max(10, n)* points of the mean square displacement), the duration, and the localization relative to the center of the synapse. Maps were obtained as done for the beads map (using a gaussian kernel).

### Antigen-stripping experiments

Round glass coverslips were coated with anti-HEL antibody by overnight incubation. The coverslips were washed with PBS and imaged to obtain the control image. They were then placed on the antigen-coated PAA gel for 30 s to 1 minute. They were stripped off the surface of the gel and imaged as soon as possible using laser scanning microscope (Leica) with a  40x 1.4 NA oil immersion objective. Fluorescence on these images indicated the presence of detached antigen. Absence of fluorescence in stripped gel suggests that quenching is not due to the presence of many layer of antigen.

### Antigen extraction quantifications

Movies of fluorescent antigen were quantified by measuring the fluorescence intensity in the cell *S(t)*, subtracting the initial point *S(0)* and dividing by *S(0)*. So the plotted quantity (*S*(*t*)–*S*(0))/*S*(0) represents the relative increment in fluorescence. When compared with the energy, this quantity signal was further divided by the maximum of the signal to compare the trend in the curve.

### TEM: energy and flux

The traction force algorithm was based on that used by Butler et al.^52^ and modified by Mandal et al.^15^. Force reconstruction was conducted with the assumption that the substrate is a linear elastic half space, using Fourier Transform Traction Cytometry with Tikhonov regularization (regularization parameter was set to 5 × 10^−19^). The position of the beads in reference image and deformed one was measured using MTT algorithm^53^. The problem of calculating the stress field from the displacement is solved in Fourier space then inverted back to real space. The final stress field is obtained on a grid with 0.432 µm spacing (four pixels). All calculations and image processing were performed in Matlab.

Given the size of B cells, the density of beads, the magnitude of displacement, some parameters needed optimization for the analysis. In particular for the detection algorithm (MTT): search window size (5 pixels), particle radius (2.5 pixels), and maximum distance for nearest neighbor (four pixels). Pixel size of spinning disk confocal microscope is 108 nm (we occasionally used another setup with pixel size 160 nm, but the parameters did not need adjustment). Same parameters were applied for noise detection by measuring force in a non-stressed area not too far from the cell. A quality check of the TFM algorithm is given by the non-equilibrated forces, i.e., by the ratio of the sum of forces vectors (which should be zero) to the sum of magnitude of the forces. Lower ratio signifies higher quality of the analysis. We checked that all analyzed data this ratio was below 0.15. Further calculations based on the output of the algorithm were performed to extract the total strain energy (defined as the sum over the entire cell area of the scalar product force by displacement). Fluxes were calculated by standard vector analysis (Green’s theorem): the flux is the integral over the cell area of the divergence of the 2D field (displacement). An outward flux represents pushing forces and inward flux represents pulling forces.

Note that even if in theory the forces are supposed to be zero outside the cell, we decided not to introduce this constraint to avoid border effects. However, when we compute energy and fluxes, we use the mask of the cell extracted by using an ImageJ custom-made macro. The mask was increased by 10% (dilation of the binary image using Matlab morphological tools) to avoid excessive cropping of the force/displacement field.

In order to respect physiological rigidity, the Young modulus of the gel is *E* ~ 500 Pa. This which limits the number of particles that can be inserted within the gel without altering its properties and prevented us to use more resolutive methods^54,55^. However, our setup has the advantage of being relatively simple to implement on classical confocal microscopy keeping a relatively good resolution. The imaging conditions in soft gel are also the reason for a poor point spread function which prevented us to implement 2.5D force measurements as done by Legant et al.^56^. This is also the reason for a statistical treatment of the *z* displacement in the 3D tracking experiement (see the quantification of the std(*z*) in Fig. 5a).

### TFM algorithm for coordinated and non-coordinated forces

We determined whether a bead belongs to the coordinated or non-coordinated group, by calculating the mean correlation between the displacement vector associated with the bead and its nearest neighbors (within 1 µm range). Beads with a correlation coefficient below 0.5 were considered to belong to the non-coordinated pool. Note that we define a correlation that does not depend on the magnitude of the displacement vectors but only of their relative orientation. This implies that beads moving a little or not at all have low correlations coefficient and build up the non-coordinated pool.

### Spectral analysis

To extract a typical time-scale of the collective pulsatile dynamics (Fig. 3f) the coordinated energy was first de-trended subtracting the background obtained smoothing the original signal with a low pass filter (Savitsky–Golay filter with third degree polynomial and a window of 500 s, 101 frames). The filter was run a second time to eliminate high frequency noise (Savitsky–Golay filter with third degree polynomial and a window of 50 s, 11 frames). The power spectrum was then computed on the de-trended signal using maximum entropy algorithm (Matlab). The maximum (if present) was selected in frequencies between 1/50 Hz and 1/500 Hz (to avoid effects introduced by the smoothing).

### *Z* movement measurement

B cells from MD4 mice were settled onto a PAA gel coated with HEL. Cells were allowed to settle for 10 min before imaging with an inverted spinning disk confocal microscope (Eclipe Ti Nickon/Roper Spinning head) equipped with a × 40 water immersion objective 1.4 NA and a CoolSNAP HQ2 camera (pixel size 6.4 µm) with Metamorph software (Molecular Device, France). Stacks of 16 images were taken with dz = 0.2 µm, every 6 s for 60–360 s. We performed 3D single particle tracking (Trackmate, Fiji) and analyzed the trajectory in Matlab. We analyzed and plotted the standard deviation of the *z* position in subtrajectories of 10 frames (to pull together the movies that have different lengths). Center and radius of the cell were extracted from the mask and used to compute the “normalised position” of the trajectory in the average cell. The central region is considered having a radius *r* = 2/3*(cell radius).

### Fluorescence-activated cell sorting antibodies

Cells were blocked with rat anti-mouse CD16/CD32 (BioLegend) and stained with: LIVE/DEAD™ Fixable Aqua Dead Cell (ThermoFischer), PerCpCy5.5 Rat anti-mouse IgD (BioLegend), Pacific Blue Rat anti-mouse B220 (BioLegend), PE Cy7 Hamster anti-mouse CD95 (BD Biosciences), PE Rat anti-mouse T- and B-cell activation antigen (BD Biosciences). Samples were attained on BD LSRFortessa X20 and analyzed using FlowJo software.

### Mice immunization

HEL (Sigma)-OVA (Sigma) coated beads used in immunization experiments were prepared as follows: 7.5 µg of biotinylated HEL + 7.5 µg of biotinylated OVA 647 were incubated overnight at 4 °C with 10^7^ streptavidin-coated 200 nm beads (Sigma) in 500 µl, washed four times and re-suspended in PBS–BSA 1% at a concentration of 80 × 10^6^ beads/µl. Mice were injected subcutaneously in the left flank with 50 µl beads in Alum (ThermoScientific) in a ratio 1:1 (mice received 4 × 10^9^ beads or 3 µg HEL + 3 µg OVA). Draining (inguinal) lymph nodes were collected on day 14.

### Lymph node immunofluorescence

Eight micrometer-thick lymphnode sections were blocked for 30 minutes with 5% goat serum (DakoCytomation) in PBS. GL7 antibody was incubated overnight at 4 °C, followed by washing with PBS and staining of the remaining directly conjugated antibodies for 1 h at room temperature. The following antibodies were used: Alexa Fluor 488-conjugated rat anti-mouse T and B-cell activation antigen (BioLegend) and PE-conjugated rat anti-mouse B220 (BioLegend), Alexa Fluor 647-conjugated anti-mouse CD169 (BioLegend). Afterwards, the tissue sections were washed with PBS and mounted with Prolong Diamond mounting medium (Invitrogen). Images were collected using a confocal microscope (Zeiss LSM880) and analyzed using ImageJ software.

### Statistics

All graphs and statistical analyses were performed with GraphPad Prism 5 (GraphPad Software) and MATLAB. In most cases non-parametric test (Mann–Whitney) test was used to determine statistical significance unless otherwise stated. Bar graphs show the median ± interquartile range (IQR) or mean ± standard error mean (SEM). Graphs representing strain energy and displacement flux were aligned to start at time zero, dot plots of strain energy show the average of each cell at the plateau.

### Reporting summary

Further information on research design is available in the Nature Research Reporting Summary linked to this article.

## Supplementary information

Supplementary Information

Peer Review File

Supplementary Movie 1

Supplementary Movie 2

Supplementary Movie 3

Supplementary Movie 4

Supplementary Movie 5

Supplementary Movie 6

Reporting Summary

Supplementary Movie 7

Data Source file

## Data Availability

The raw and treated data are available from the corresponding authors upon request. The source data underlying Figs. 1d–i, 3a–b, 3d, 4b–d, 4g–h, 5a–b, 5e–f, 6c, 7b–d, 7f, 7i, 8a, 8d–i and Supplementary Figs 1b–c, 2a–b, 3c and 6b are provided as a Source Data file.

## Code availability

TFM analysis codes (Matlab) and image quantification tools (ImageJ and Matlab) are available from the corresponding authors upon request.
