# Supplementary material for: Actomyosin-driven force patterning controls endocytosis at the immune synapse
Source: Nat Commun. 2019 Jun 28;10:2870. doi: 10.1038/s41467-019-10751-7 (PMC6599028; doi:10.1038/s41467-019-10751-7)
Supplement: Supplementary file 1 — Supplementary Information [file 41467_2019_10751_MOESM1_ESM.pdf]

Actomyosin-driven force patterning controls endocytosis at the immune synapse

SUPPLEMENTARY INFORMATION

Anita Kumari[1,2$], Judith Pineau[1,2$], Pablo J. Sáez[1], Mathieu Maurin[1], Danielle Lankar[1], Mabel San Roman[1], Katharina Hennig[3], Vanessa F. Boura[4], Raphael Voituriez[5], Mikael C.I. Karlsson[4], Martial Balland[3], Ana-Maria Lennon Dumenil[1,*], Paolo Pierobon[1,*]

[1]Institut Curie, PSL Research University, INSERM U932, 26 rue d'Ulm, 75248 Paris Cedex 05, France.

[2]Université Paris Descartes, Paris, France

[3]Laboratoire Interdisciplinaire de Physique, Université Joseph Fourier (Grenoble 1), 38402 Saint Martin d'Hères Cedex 9, France.

[4]Department of Microbiology, Tumor and Cell Biology, Karolinska Institutet, Stockholm, Sweden.

[5]Laboratoire de Physique Théorique de la Matière Condensée, UMR 7600 CNRS /UPMC and Laboratoire Jean Perrin, UMR 8237 CNRS /UPMC, 4 Place Jussieu 75255 Paris Cedex 05, France

$ Equal contribution

* Corresponding authors

Email: ana-maria.lennon@curie.fr, paolo.pierobon@curie.fr

Actomyosin-driven force patterning controls endocytosis at the immune synapse
Supplementary notes

1 Analysis of the coordinated and non-coordinated bead movements

To distinguish between coordinated and non-coordinated bead movements we analyse each beads according to the degree of correlation with their neighbours. Each bead i is displaced of a vector $\vec{v_i} = (dx_i, dy_i)$. So for a chosen bead j we compute the correlation coefficient r_j with the N nearest neighbours belonging to region C as:

$$r_j = \frac{1}{N} \sum_{i \in C} \frac{\vec{v_i} \cdot \vec{v_j}}{v_i v_j}$$

where C is the circle r radius $1 \mu m$ around bead j; this is the distance allowing to average on a mean of 5 beads. This index is equivalent to the average cosine of the angle formed by the displacements (in analogy to liquid crystal order parameter):

$$r_j = \frac{1}{N} \sum_{i \in C} \cos(\theta_{i,j}) = \langle \cos(\theta_{i,j}) \rangle_{i \in C}$$

At each time point and for each beads i the value r_i is computed and the beads classified according to:

$$\begin{cases} r_j < 0.5, & \text{bead } j \text{ non-coordinated} \\ r_j \geq 0.5 & \text{bead } j \text{ coordinated} \end{cases}$$

As can be deduced from the definition, r_j does not take into account the magnitude of displacement. Beads that, because of noisy detection and microscopy acquisition, show small apparent displacements will be included in this pool because the orientations of their displacement vectors are randomly distributed. This is the reason for the number of non-coordinated beads decreasing in Supp.Fig.2b.

2 Underestimation of the non-coordinated pool of forces

The non-coordinated displacements results of a force with a component perpendicular to the substrate. The traction force algorithm used in this work solves the 2D problem. In this paragraph we estimate the error we might make applying the TFM algorithm on a displacement that in reality results from a point-like force $\mathbf{F} = (0, 0, F)\delta(x)\delta(y)\delta(z)$. This force, perpendicular to the gel and exerted from a protrusion, (but the argument works also for outward forces, changing the sign), will generate a displacement $\mathbf{u} = \mathbf{G_3} \cdot \mathbf{F}$ where $\mathbf{G}_3$ is the 3D Boussinesq Green function:

$$\mathbf{G}_3 = \frac{1}{4\pi E R^3} \begin{bmatrix} R^2 + x^2 & xy & xz \\ xy & R^2 + y^2 & yz \\ xz & yz & R^2 + z^2 \end{bmatrix}$$

where $R^2 = x^2 + y^2 + z^2$. The displacement reads:

$$\mathbf{u} = (dx, dy, dz)^t = \frac{F}{4\pi E R^3} \left(xz, yz, R^2 + z^2\right)^t$$

(where the t apex indicate transposed vectors). However, we measure only $\tilde{\mathbf{u}} = (dx, dy)^t$, hence the apparent components measured by TFM algorithm is $\tilde{\mathbf{F}} = \mathbf{G}_2^{-1} \cdot \tilde{\mathbf{u}}$ where

$$\mathbf{G}_2 = \frac{1}{4\pi E r^3} \begin{bmatrix} r^2 + x^2 & xy \\ xy & r^2 + y^2 \end{bmatrix}$$

where $r^2 = x^2 + y^2$ indicate the position where we measure the displacement. Simplifying the equation we are left with

$$\tilde{\mathbf{F}} = \mathbf{G}_2^{-1} \cdot \tilde{\mathbf{u}} = \frac{rzF}{2R^3}(x, y)^t$$

The ratio between force magnitude will give the percentage of underestimation:

$$\frac{|\tilde{\mathbf{F}}|}{|\mathbf{F}|} = \frac{\sqrt{\tilde{F}_x^2 + \tilde{F}_y^2}}{F} = \frac{r^2 z}{2(r^2 + z^2)3/2}$$

This function has a maximum in $r = z\sqrt{2}$ but is close to zero for small r, making our estimation completely wrong. The value of the maximum is independent of z and is $3^{-3/2} \approx 20\%$ meaning that at our best we underestimate the real force of a factor 5 (only when the displacement is measure close to the application point). For this reason we cannot rely on the TFM algorithm for 3D analysis. However, displacement and number of beads are pure observations and a comparison between the two populations is meaningful.

3 Myosin II driven pulsatile contractions can lead to central patterns

In Reference [3] a model for cell motility is proposed where the velocity of the cell is coupled to the displacement of polarity cues via an actomyosin flow. Inspired by this model we propose that the pulsatile dynamics of actomyosin can transport membrane bound molecules towards the center of the synapse and and therefore help concentrating the endocytic machinery there. However the formation of a pattern, such as the accumulation of molecules in the center, is guaranteed only for certain range of parameters. Here we investigate whether in which condition actomyosin pulsatile contraction can generate patterns.

Let us consider a molecular species having concentration $c(\mathbf{r}, t)$ at the point $\mathbf{r}$ at instant t. We assume that, because of direct or indirect interactions with actin filaments, it is advected by the actin flow of speed $\mathbf{V}(t)$ and denote by D the diffusion coefficient. Its dynamics then follows

$$\partial_t c(\mathbf{r}, t) - \nabla \cdot [\mathbf{V}(t) c(\mathbf{r}, t)] = D \nabla^2 c(\mathbf{r}, t) \tag{1}$$

In our case $\mathbf{V}$ is time independent and we assume it is switched on/off with rate k_1, k_0 (according to a random telegraph process). For the sake of simplicity, we will assume below that the actin flow is centripetal and uniform in space, so that it can be written $\mathbf{V} = -V(t)\mathbf{u}_r$ where $\mathbf{u}_r$ is the radial unit vector. Under these assumptions eq. 1 can be rewritten as a system of partial differential equations that describe the dynamics of the two populations c_1 and c_0 of particles subject or not to advection (respectively). The velocity is now time independent:

$$\begin{cases} \partial_t c_0 = D\nabla^2 c_0 - k_1 c_0 + k_0 c_1 \\ \partial_t c_1 = D\nabla^2 c_1 + \nabla \cdot [c_1 \mathbf{V}] + k_1 c_0 - k_0 c_1 \end{cases} \tag{2}$$

This system of linear differential equations can be solved explicitely for centripetal flows; it is however instructive to consider an adiabatic approximation where the typical timescales for diffusion time and advection are larger than the on-off kinetics of the velocity:

$$k_1^{-1}, k_0^{-1} \ll \frac{L}{V}, \frac{L^2}{D}. \tag{3}$$

This is justified since typical on/off times are $k_1^{-1} < k_0^{-1} \leq 2min$, the size of the cell is $L \approx 5\mu m$ and diffusion is $D \approx 0.05\mu m^2/s$, which gives $L/V \approx 5min$ and $L^2/D \sim 10min$.

In this case the concentrations equilibrate through the on/off kinetics faster than through the other processes and therefore one can assume $c_0/c_1 = k_1/k_0$ and substitute:

$$c_1 = \frac{k_0}{k_1 + k_0}(c_1 + c_0) = \alpha c$$

summing the two equations in eq.2 and integrating, one obtains for the total concentration $c = c_1 + c_0$:

$$D\nabla^2 c + \nabla \cdot \mathbf{V}\alpha c = 0$$

In cylindrical coordinates (and using the equation for the radial component (the system is centrosymmetric) reads:

$$(D\nabla^2 c + \nabla \cdot \mathbf{V}\alpha c)_r = \frac{1}{r}\frac{\partial}{\partial r}\left(r\frac{\partial c}{\partial r}\right) + \alpha V \frac{1}{r}\frac{\partial c}{\partial r} = 0$$

We solve this equation with open boundary conditions:

$$D\frac{\partial c}{\partial r} + \alpha V c = 0$$

which gives the usual exponential solution:

$$c = c_0 e^{-\alpha V x/D}.$$

Clearly this defines a length scale $\lambda = D/(\alpha V)$ that can be used as a criterion for the formation of a pattern: when is smaller than the radius of the synapse $\lambda \ll L$ then the pattern is formed and there exists an appreciable gradient along the radius. Note that approximation in eq. 3 simplifies the problem but it is not necessary for its solution: being linear the stationary solution will be anyway exponential and the relevant length would be λ as above.

Pluging in experimental numbers:

- the diffusion constant of a molecule attached to the membrane is $D \leq 1\mu m^2/s$ however for BCR this is typically $D \approx 0.05\mu m^2/s$
- flow speed is $V \approx 1\mu m/s$ from rough particle image velocimetry measurements on our data (in [1] a peak value 5 times higher is presented on stiff surfaces)
- α can be estimated observing that the typical time of a pulsation is few frames ($k_1 = \tau_1^{-1} \approx 15s$) and the typical pulsation is around $170s$ ($\tau_1 + \tau_0 = k_1^{-1} + k_0^{-1} \approx 120s$): then $\alpha = \frac{\tau_1}{\tau_1 + \tau_0} \approx 0.09$

we find indeed a typical length scale in the problem of:

$$\lambda = \frac{D}{\alpha V} \approx 0.56\mu m$$

which means that the species are localised in the center provided that the diffusion is low enough (which is particularly true for membrane bound species, indeed D can be even smaller, for BCR this is typically $D_{mem} = 0.05\mu m^2/s$). This means that the pattern will be destroyed on a timescale of $L^2/D \approx 500s$. Typical pulsations have shorter typical timescales, suggesting that this could be a mechanism to gather molecules in the center of the synapse.

4 Force required for antigen extraction

In this section we give an estimation of the load on each HEL molecule based on our measurements. We first measured the concentration of HEL on gel using two techniques:

1. count of secondary antibodies fluorescence by using photobleaching and Poisson statistics as done in [5]; this gives about 12 fluorophores/μm^2 which might means (assuming 1 fluorophore/secondary antibody, 2 primary antibodies/HEL and 2 secondary per primary) around maximum 50 HEL molecules/μm^2.

2. measuring the amount of antigen not attached to the gel by Nanodrop and comparing with the original concentration. We found that when coating a gel with $100\mu g/ml$ solution of HEL ($7\mu M$), 30% of the proteins are retained, hence using 200µl (i.e. $\sim 20\mu g = 1.4nMol = 8.4 \cdot 10^{14} molecules$) the gel retains $2.8 \cdot 10^{14} molecules$. The mesh size of a $500Pa$ gels is about $50 - 100nm$ and this would allow the HEL molecule ($\sim 5nm$) to go through the gel and functionalise it even in bulk. The surface of the gel is $A = 250mm^2$ and its volume $V = 25mm^3$ we obtain that there are roughly $11000 molecules/\mu m^3$ or at the surface there are $c^{2/3} = 500 molecules/\mu m^2$.

We deduced that HEL concentration is in the range $50 \sim 500 molecules/\mu m^2$. We chose (cautionary) to consider the smallest value. The shear stresses we measure in our experiments are maximum $80Pa$, hence a force of $80pN$ distributed over $1\mu m^2$. If all the forces were equally distributed among the HEL molecules one expects on average less than $2pN$ per molecule which

make statistically difficult for shear force to contribute to antigen detachment. In contrast point like forces are necessary localised and reach values that could provide detachment of a single molecule. In fact mechanics is required for detachment forces over $16pN$ ([6]) and the force required to break the covalent bond between HEL and Polyacrylamide is possibly of the order of $1nN$ (similar large forces have been observed and explained in single acto-myosin contractile elements made up of several tens of molecular motors [2]).

5 Affinity discrimination and energy scales

We suggest also that the force patterning might play a role in improving affinity discrimination, in particular in reducing the noise in the center of the synapse. Why should be this important to isolate mechanically the region where the antigen is extracted?

In [4] the authors propose that the affinity discrimination is mechanical, this implies that the cell has to pull on the antigen to overcome an energetic barrier of height ΔU. The affinity depends essentially on the dissociation constant which scales exponentially with the energy:

$$K_D \sim e^{-\Delta U/k_B T}$$

(where $k_B T$ $4pN.nm$, the scale of thermal fluctuations at equilibrium).

A N folds increase in affinity between antigen 1 and 2 with two energetic barrier ΔU_1 and ΔU_2 can be written as:

$$N = \frac{k_1}{k_2} = \frac{e^{-\Delta U_1/k_B T}}{e^{-\Delta U_2/k_B T}} = e^{-(\Delta U_1 - \Delta U_2)/k_B T}$$

This results in an energy barrier difference of:

$$(\Delta U_1 - \Delta U_2) = (\ln N) k_B T$$

In the case of antigens used in [4] $N = 10$ and the energy difference is of the order of $ln(10)k_B T \approx 2.3 k_B T$. As a term of comparison the maximal work that a single Myosin II molecule can exert is $2.5 k_B T$. Therefore the affinity discrimination mechanism could be easily disturbed by application of external forces or even weak mechanical noise. The peripheral stress could serve precisely to seal the synapse and mechanically isolate it from external noise.

Supplementary References

[1] C. Liu, H. Miller, G. Orlowski, H. Hang, A. Upadhyaya, and W. Song. Actin reorganization is required for the formation of polarized B cell receptor signalosomes in response to both soluble and membrane-associated antigens. *Journal of immunology (Baltimore, Md. : 1950)*, 188(7):3237–46, apr 2012.

[2] J. Lohner, J.-F. Rupprecht, J. Hu, N. Mandriota, M. Saxena, J. Hone, D. P. de Araujo, O. Sahin, J. Prost, and M. Sheetz. Myosin filaments reversibly generate large forces in cells. *bioRxiv*, page 296400, apr 2018.

[3] P. Maiuri, J. F. Rupprecht, S. Wieser, V. Ruprecht, O. Bénichou, N. Carpi, M. Coppey, S. De Beco, N. Gov, C. P. Heisenberg, C. Lage Crespo, F. Lautenschlaeger, M. Le Berre, A. M. Lennon-Dumenil, M. Raab, H. R. Thiam, M. Piel, M. Sixt, and R. Voituriez. Actin flows mediate a universal coupling between cell speed and cell persistence. *Cell*, 161(2):374–386, 2015.

[4] E. Natkanski, W.-Y. Lee, B. Mistry, A. Casal, J. E. Molloy, and P. Tolar. B cells use mechanical energy to discriminate antigen affinities. Materials and Methods. *Science (New York, N.Y.)*, 340(6140):1587–1590, 2013.

[5] C. R. Nayak and A. D. Rutenberg. Quantification of fluorophore copy number from intrinsic fluctuations during fluorescence photobleaching. *Biophysical Journal*, 101(9):2284–2293, 2011.

[6] Z. Wan, X. Chen, H. Chen, Q. Ji, Y. Chen, J. Wang, Y. Cao, F. Wang, J. Lou, Z. Tang, and W. Liu. The activation of IgM- or isotype-switched IgG- and IgE-BCR exhibits distinct mechanical force sensitivity and threshold. *eLife*, 4(August):1–24, 2015.

SUPPLEMENTARY FIGURES

Supplementary figure 1

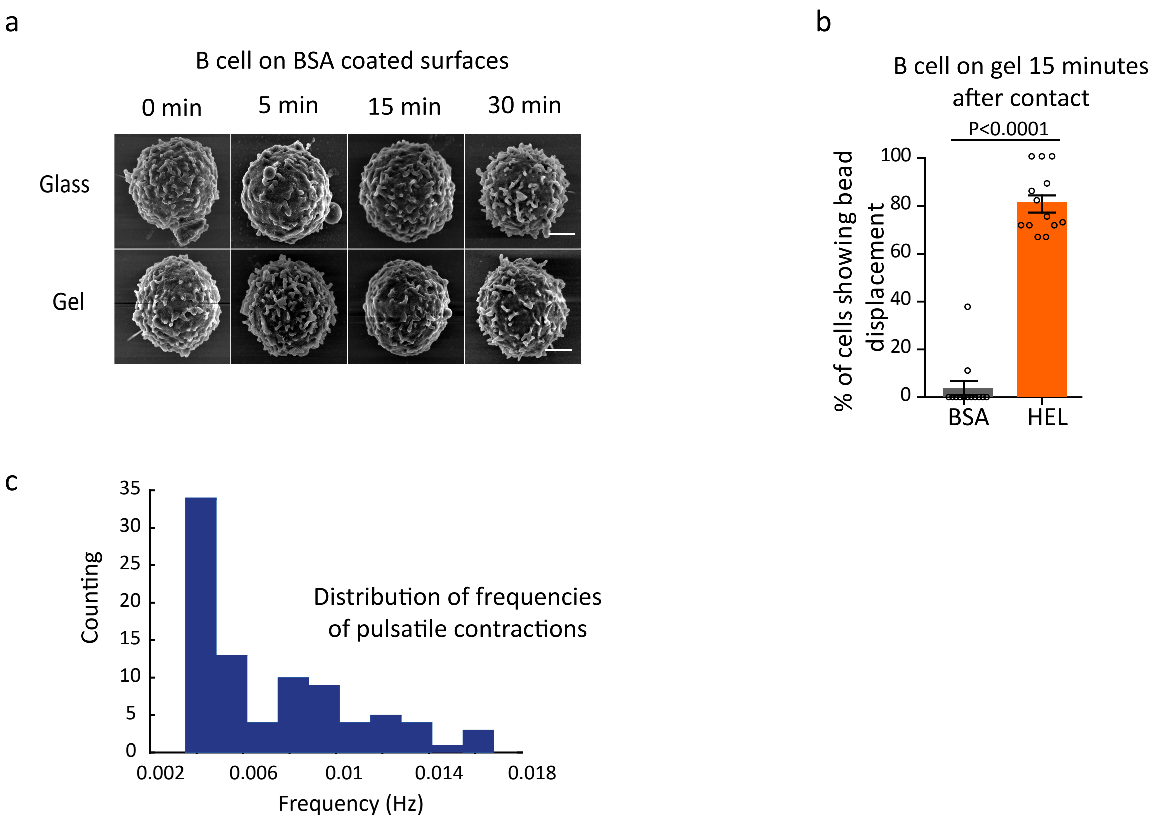

Supplementary Figure . 1: (a) Scanning electron microscopy of fixed B cells on BSA coated glass and polyacrylamide gels, scale bar is 2µm. (b) Percentage of B cells showing bead displacement in HEL and control BSA condition (n=13 mice), error bars represents mean±SEM, Mann Whitney test was performed for statistical analysis. (c) Histogram of the spectra maxima in frequency returning a median typical time of 170s for the period of global contractions (n=45). Source data are provided as a Source Data file.

Supplementary figure 2

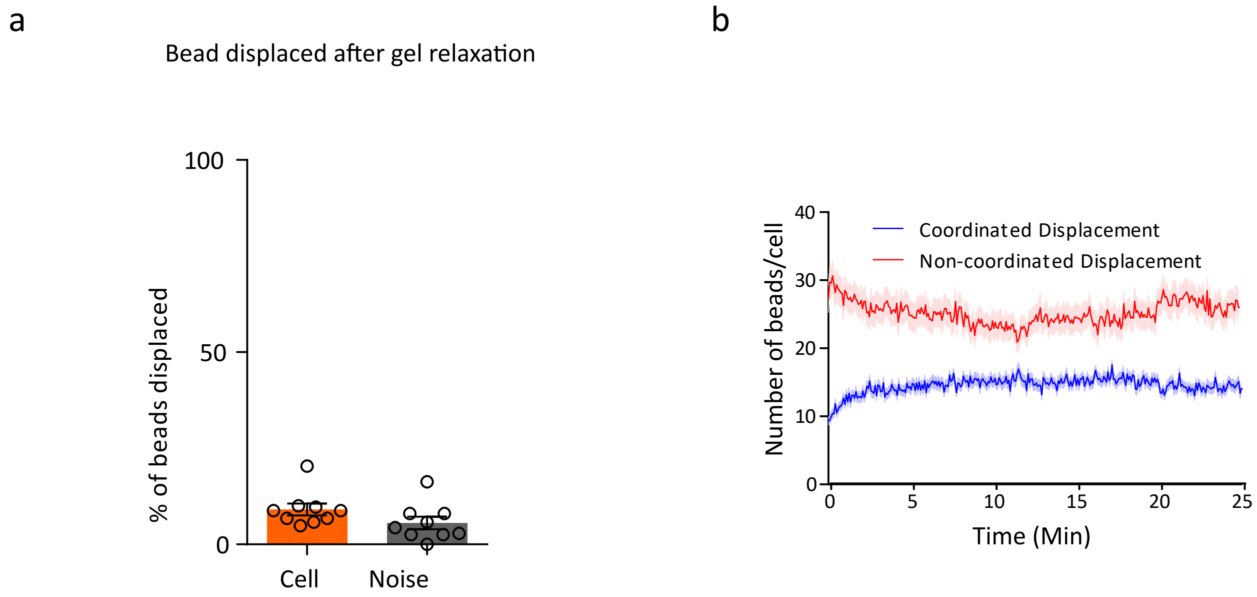

Supplementary figure 2: (a) Non-coordinated displacements are not resulting from gel deterioration: percentage of bead displaced on removing the cells after the experiments, Mean±SEM (n=15), Mann Whitney test was performed for statistical analysis. (b) Number of beads per cell over time in coordinated and non-coordinated type of forces. Error bars represent Mean±SEM. Source data are provided as a Source Data file.

Supplementary figure 3

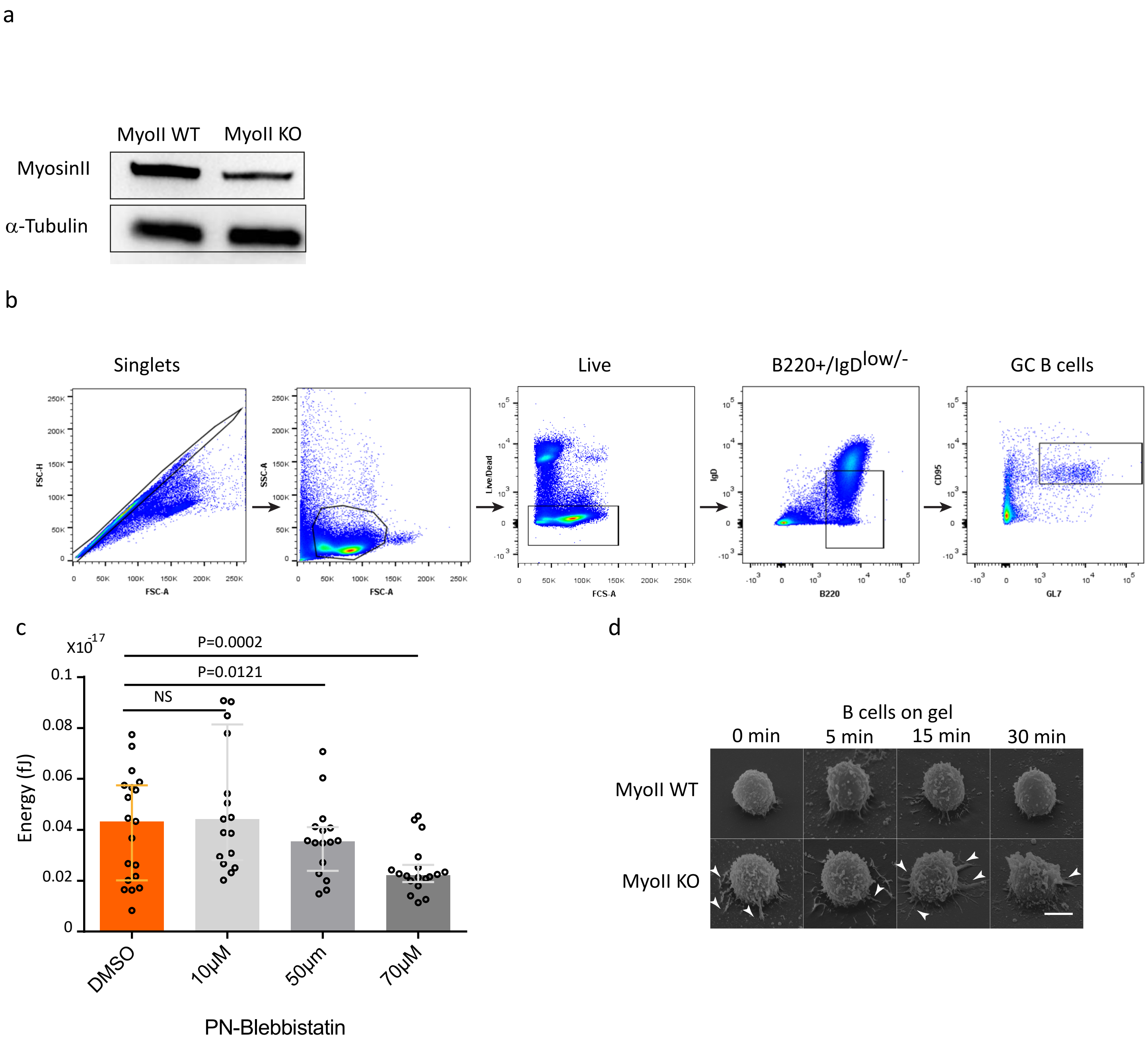

Supplementary figure 3: (a) Western blot analysis of the efficiency of Myosin II knock out, α-tubulin was used as loading control (example selected out of n=4 experiments, all showing the same tendency). (b) Gating strategy for selection of the germinal centre B cell: these cells are selected as B220+/IgD low/CD95+/GL7+. (c) Concentration dependent decrease in strain energy of para-Nitroblebbistatin treated B cells (error bars representing median±IQR, n=15, 17, 14 and 18, 3 independent experiments, 3 mice, Mann Whitney test was performed for statistical analysis). (d) Scanning electron microscopy of fixed B lymphocytes in Myosin IIA KO and WT condition, arrows showing non retracted fibers (scale bar: 5μm). Source data are provided as a Source Data file.

Supplementary figure 4

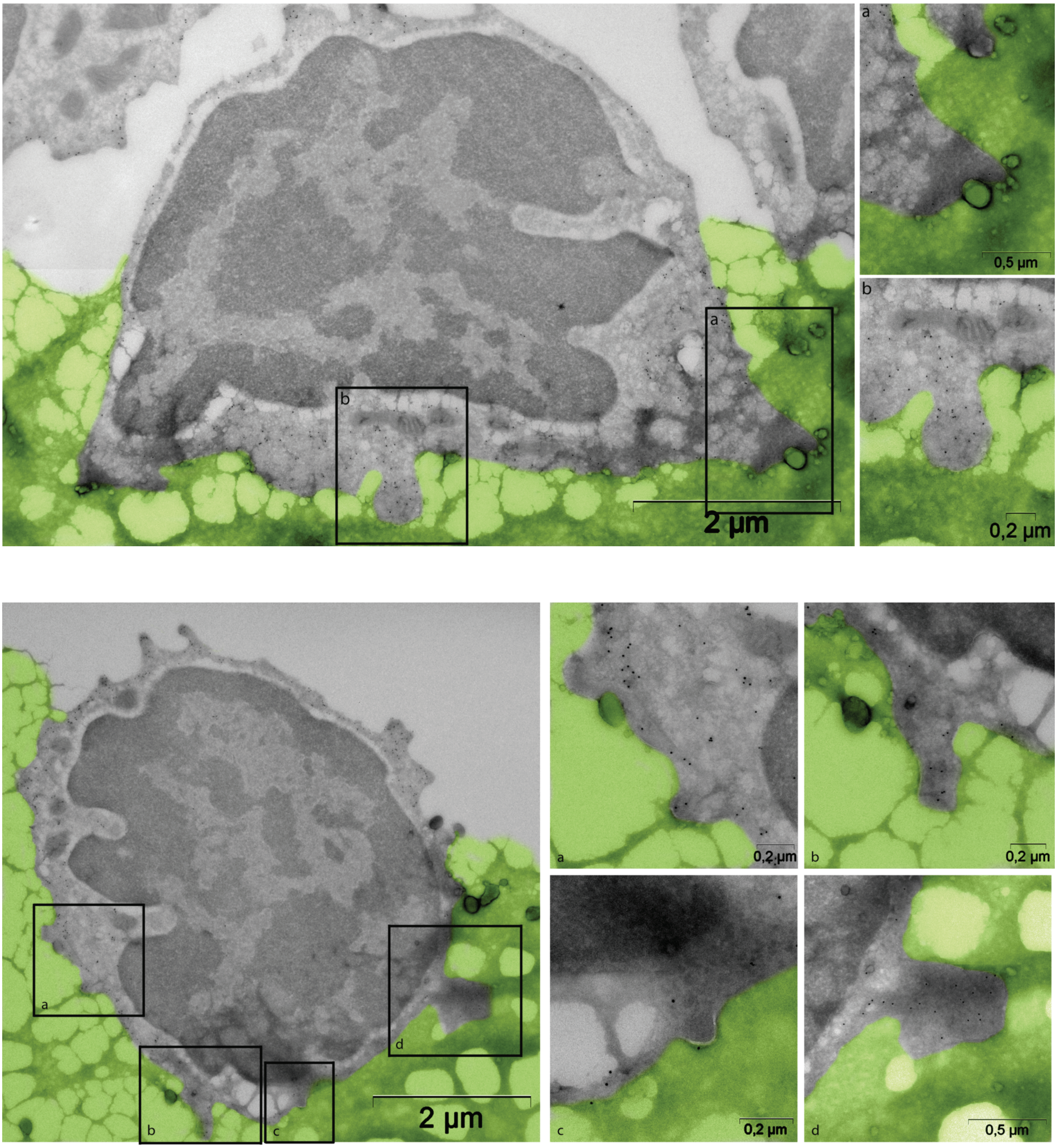

Supplementary figure 4: Transmission electron microscopy images of B cells on HEL coated polyacrylamide gels (green) showing examples of actin rich protrusions.

Supplementary figure 5

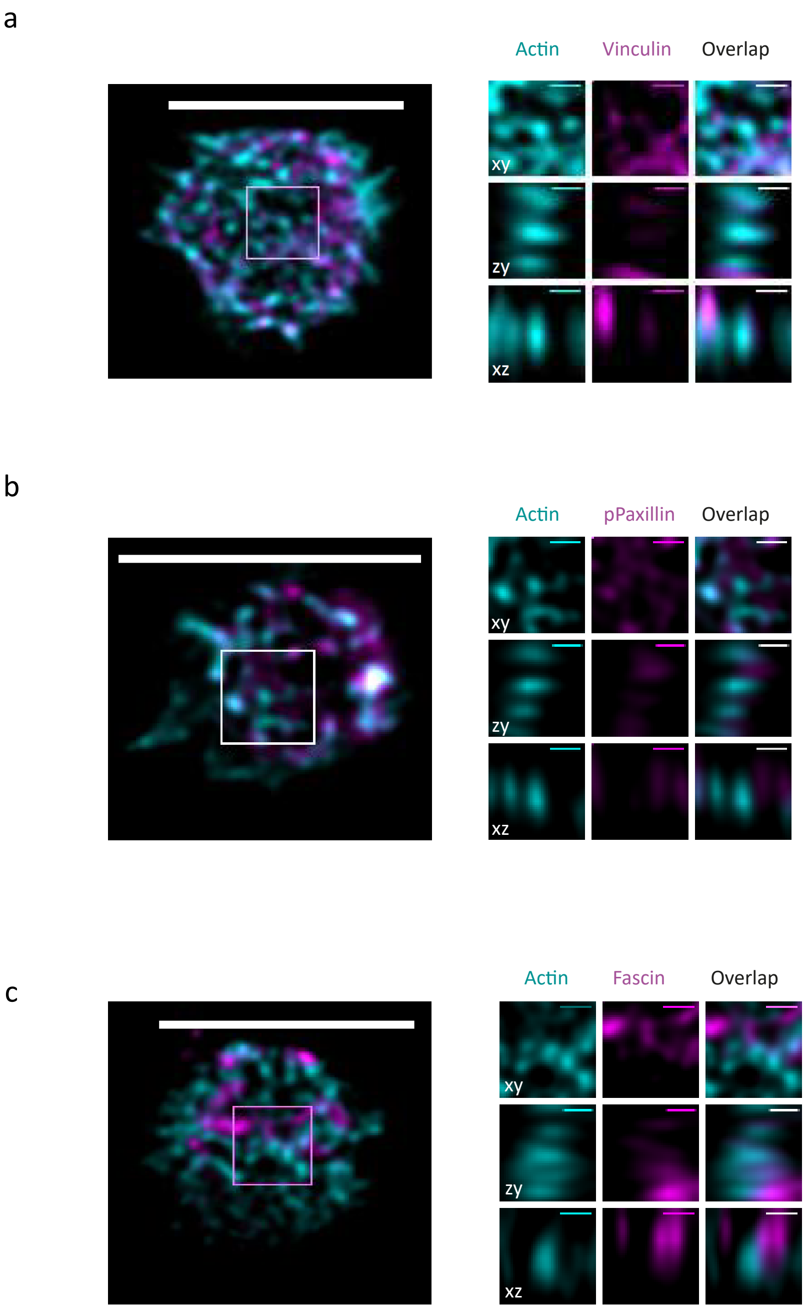

Supplementary figure 5: Immunostaining of actin (cyan) and (a) vinculin (magenta), (b) phospho-paxillin (magenta) or (c) fascin (magenta) and zoomed orthogonal projections on different planes (scale bar 5µm, zoom 0.5µm); images show no colocalization.

Supplementary figure 6

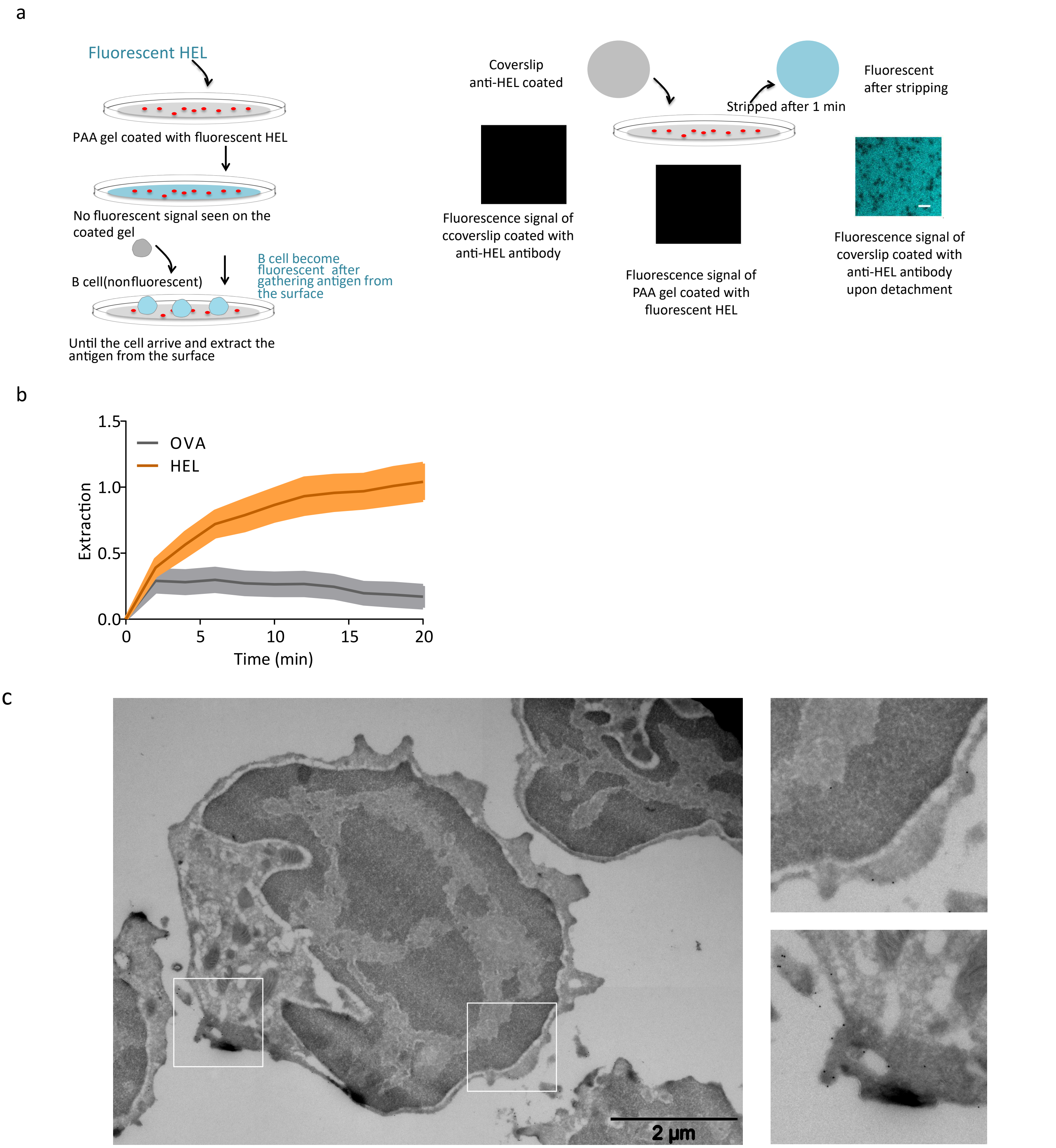

Supplementary figure 6: (a) Scheme showing stripping experiments: anti-HEL coated coverslip (non-fluorescent) was put 1 minute in contact with fluorescent HEL coated gel (non-fluorescent) and successively stripped; the coverslip becomes fluorescent (scale bar is 2 µm). (b) Antigen extraction is specific: fluorescent HEL intensity upon B cell contact compared to fluorescent Ova, error bars represent Mean±SEM (n=67 Antigen, n=67 Ova, 2 independent experiments). (c) Transmission electron microscopy images of myosin II KO B cells striped from HEL coated gels 30 minutes after contact: the beads show HEL mainly at the surface of the cell. Source data are provided as a Source Data file.
