# Supplementary material for: Actomyosin-driven force patterning controls endocytosis at the immune synapse
Source: Nat Commun. 2019 Jun 28;10:2870. doi: 10.1038/s41467-019-10751-7 (PMC6599028; doi:10.1038/s41467-019-10751-7)
Supplement: Supplementary file 9 — Reporting Summary [file 41467_2019_10751_MOESM9_ESM.pdf]

nature research

Corresponding author(s): DBPR NCOMMS-19-09577-A

Last updated by author(s): May 23, 2019

Reporting Summary

Nature Research wishes to improve the reproducibility of the work that we publish. This form provides structure for consistency and transparency in reporting. For further information on Nature Research policies, see Authors & Referees and the Editorial Policy Checklist.

Statistics

For all statistical analyses, confirm that the following items are present in the figure legend, table legend, main text, or Methods section.

n/a	Confirmed	
☐	☒	The exact sample size (n) for each experimental group/condition, given as a discrete number and unit of measurement
☐	☒	A statement on whether measurements were taken from distinct samples or whether the same sample was measured repeatedly
☐	☒	The statistical test(s) used AND whether they are one- or two-sided<br>*Only common tests should be described solely by name; describe more complex techniques in the Methods section.*
☒	☐	A description of all covariates tested
☒	☐	A description of any assumptions or corrections, such as tests of normality and adjustment for multiple comparisons
☐	☒	A full description of the statistical parameters including central tendency (e.g. means) or other basic estimates (e.g. regression coefficient) AND variation (e.g. standard deviation) or associated estimates of uncertainty (e.g. confidence intervals)
☐	☒	For null hypothesis testing, the test statistic (e.g. F, t, r) with confidence intervals, effect sizes, degrees of freedom and P value noted<br>*Give P values as exact values whenever suitable.*
☒	☐	For Bayesian analysis, information on the choice of priors and Markov chain Monte Carlo settings
☒	☐	For hierarchical and complex designs, identification of the appropriate level for tests and full reporting of outcomes
☒	☐	Estimates of effect sizes (e.g. Cohen's d, Pearson's r), indicating how they were calculated

Our web collection on statistics for biologists contains articles on many of the points above.

Software and code

Policy information about availability of computer code

Data collection	See material and methods. Microscopy data were collected through Metamorph (spinning disk data) and LAS X (Leica SP8). Raw data are available on request.
Data analysis	Images for TFM analysis were registered and cropped with Fiji (ImageJ). TFM analysis was carried out with a custom Matlab code described in the materials and methods. Fluorescence data were analyzed through Fiji and the quantification elaborated with Matlab. The codes are not provided with the manuscript because not (yet) easily readable and commented but are available "as they are" on request.

For manuscripts utilizing custom algorithms or software that are central to the research but not yet described in published literature, software must be made available to editors/reviewers. We strongly encourage code deposition in a community repository (e.g. GitHub). See the Nature Research guidelines for submitting code & software for further information.

Data

Policy information about availability of data

All manuscripts must include a data availability statement. This statement should provide the following information, where applicable:

- Accession codes, unique identifiers, or web links for publicly available datasets
- A list of figures that have associated raw data
- A description of any restrictions on data availability

As stated in the paper all raw data are available from the corresponding authors on request. Source data are available online as Source Data file.

Field-specific reporting

Please select the one below that is the best fit for your research. If you are not sure, read the appropriate sections before making your selection.

☒ Life sciences ☐ Behavioural & social sciences ☐ Ecological, evolutionary & environmental sciences

For a reference copy of the document with all sections, see nature.com/documents/nr-reporting-summary-flat.pdf

Life sciences study design

All studies must disclose on these points even when the disclosure is negative.

Sample size	No particular method has been used for the choice of the sample size. For most of experiments we chose to repeat experiments at least 3 times per condition and the number of cells available in the experiment has been maximized compatibly with the experimental setup (microscopy time, camera capabilities and cell survival).
Data exclusions	TFM data where the measurements on unperturbed part of the gel were found to give a positive contractile energy (due to focus loss or other experimental problems) were excluded from the analysis as the acquisition was considered too noisy.
Replication	Experiments on gels were optimized for best reproducibility in the setup phase of the project. Analysis parameters were optimized in publication by Mandal et al , Nat. Commun. 5, 5749 (2014). All antibodies used in blot, immunofluorescence and FACS where previously tested with proper controls either here or in previous publication (cited when is the case).
Randomization	NA
Blinding	NA

Reporting for specific materials, systems and methods

We require information from authors about some types of materials, experimental systems and methods used in many studies. Here, indicate whether each material, system or method listed is relevant to your study. If you are not sure if a list item applies to your research, read the appropriate section before selecting a response.

Materials & experimental systems

n/a	Involved in the study
☐	☒ Antibodies
☒	☐ Eukaryotic cell lines
☒	☐ Palaeontology
☐	☒ Animals and other organisms
☒	☐ Human research participants
☒	☐ Clinical data

Methods

n/a	Involved in the study
☒	☐ ChIP-seq
☐	☒ Flow cytometry
☒	☐ MRI-based neuroimaging

Antibodies

Antibodies used	See Materials and Methods.
Validation	See Materials and Methods. All the antibodies were commercially available and properly validated by the company for IF/FACS/ Blots.

Animals and other organisms

Policy information about studies involving animals; ARRIVE guidelines recommended for reporting animal research

Laboratory animals	See Materials and methods. Briefly, B cells were derived from the spleen of MD4 transgenic mice (obtained in the C57BL/B6 background). Mice with a conditional deletion of myosin II in B cells were generated by backcrossing mice carrying a floxed myosin II allele (MyosinIIflox/flox) with mice expressing the Cre recombinase under the control of the CD21promoter (CD21 cre +/-). Mice expressing the hen egg lysozyme (HEL)–specific MD4receptor were also crossed with mice carrying a floxed myosin II allele. Mice were crossed at an age of eight to 10 weeks, and Cre– littermates were used as WTcontrols. The transgenic MD4, Lifeact GFP and myosin II-GFP mouse lines have been described elsewhere (mat and meth refs [2], [3]). The experiments were performed on 8 to 10week-old male or female mice.
Wild animals	NA

Field-collected samples

NA

Ethics oversight

Immunization experiments carried out at the Karolinska Institute were performed according to local ethical committee guidelines (N11/13).

Note that full information on the approval of the study protocol must also be provided in the manuscript.

Flow Cytometry

Plots

Confirm that:

☒ The axis labels state the marker and fluorochrome used (e.g. CD4-FITC).

☒ The axis scales are clearly visible. Include numbers along axes only for bottom left plot of group (a 'group' is an analysis of identical markers).

☒ All plots are contour plots with outliers or pseudocolor plots.

☒ A numerical value for number of cells or percentage (with statistics) is provided.

Methodology

Sample preparation

Described in materials and methods

Instrument

BD LSRFortessa X20

Software

FlowJO

Cell population abundance

There was no sorting of cells and therefore no post-sort fractions and no information of the purity of the samples, it was just straight FACS. All the data presented are given in absolute numbers (frequency of GC B cell are around 2-14% (sample variation) out of the B220+ IgDlow/-).

Gating strategy

Described in materials and methods

☒ Tick this box to confirm that a figure exemplifying the gating strategy is provided in the Supplementary Information.
