# Supplementary material for: Actomyosin-driven force patterning controls endocytosis at the immune synapse
Source: Nat Commun. 2019 Jun 28;10:2870. doi: 10.1038/s41467-019-10751-7 (PMC6599028; doi:10.1038/s41467-019-10751-7)
Supplement: Supplementary file 2 — Peer Review File [file 41467_2019_10751_MOESM2_ESM.docx]

**Editorial Note:** This manuscript has been previously reviewed at another journal that is not operating a transparent peer review scheme. This document only contains reviewer comments and rebuttal letters for versions considered at *Nature Communications* .

REVIEWERS' COMMENTS: 

Reviewer #1 (Remarks to the Author): 

The authors have satisfactorily addressed all my concerns. I therefore recommend to accept the manuscript for publication in Nature Communications after addressing minor concerns. 

(1) I would like to make the authors aware of the erroneous references 10 and 12 in the revised manuscript. 

(2) If the authors wish to measure the actin turnover despite low signal-to-noise ratio, they could perform photobleaching experiments and compute the turnover from the tracked ROI. 


Reviewer #2 (Remarks to the Author): 

I am impressed with the revisions; I feel that the authors 
adequately addressed all my concerns.
